# Patient engagement practice within perinatal eHealth: A scoping review

**DOI:** 10.1002/nop2.1822

**Published:** 2023-05-21

**Authors:** Jennifer N. Auxier, Miriam Bender, Henna‐Riikka Hakojärvi, Anna M. Axelin

**Affiliations:** ^1^ Department of Nursing Science The University of Turku Turku Finland; ^2^ Sue & Bill Gross School of Nursing University of California Irvine Irvine USA

**Keywords:** implementation, perinatal care, technology

## Abstract

**Background:**

There is a gap in knowledge about how perinatal eHealth programs function to support autonomy for new and expectant parents from pursuing wellness goals.

**Objectives:**

To examine patient engagement (access, personalization, commitment and therapeutic alliance) within the practice of perinatal eHealth.

**Design:**

Scoping review.

**Methods:**

Five databases were searched in January 2020 and updated in April 2022. Reports were vetted by three researchers and included if they documented maternity/neonatal programs and utilized World Health Organization (WHO) person‐centred digital health intervention (DHI) categories. Data were charted using a deductive matrix containing WHO DHI categories and patient engagement attributes. A narrative synthesis was conducted utilizing qualitative content analysis. Preferred Reporting Items for Systematic Reviews and Meta‐Analyses ‘extension for scoping reviews’ guidelines were followed for reporting.

**Results:**

Twelve eHealth modalities were found across 80 included articles. The analysis yielded two conceptual insights: (1) The nature of perinatal eHealth programs: (1) emergence of a complex structure of practice and (2) practising patient engagement within perinatal eHealth.

**Conclusion:**

Results will be used to operationalize a model of patient engagement within perinatal eHealth.

## 1 INTRODUCTION

Perinatal periods are a time when new and expectant parents should receive support towards health‐related behaviours, health prevention and coaching to maintain wellness and closeness with infants (Hantsoo et al., 2018; Hawkins et al., 2019; He et al., 2021; Marko et al., 2016). Intensive, frequent, quality health behaviour coaching and counselling of new parents is important during perinatal periods to promote patient engagement and positive perinatal outcomes (Danbjørg et al., 2014; de Mooij et al., 2018; Himes et al., 2017; Kennelly et al., 2016). Perinatal patients perceive care as satisfactory when it is personalized and supports health engagement (Labrie et al., 2021; Phillippi et al., 2016).

eHealth perinatal care that focuses on involving patients has the potential to improve quality of care (van den Heuvel et al., 2018). New models of perinatal care are emerging that show promise for benefiting perinatal patients, these aim to improve participation and collaboration between families and professional care providers (de Mooij et al., 2018; Nelson & Holschuh, 2021). Few studies have investigated how eHealth programs support parents to engage throughout the entire perinatal continuum. Perinatal care has been historically fragmented between services for maternity and neonatal care, which has limited parents' ease in engaging (Molenaar et al., 2018). Integrated eHealth systems might support the harmonization between maternity and neonatal care programs, which might be the bridge that leads to more patient participation. Examining patient engagement practices within perinatal eHealth could illuminate ways for integration of programs that are engaging, personalized and less fragmented between maternity and neonatal care.

In 2015, the World Bank Group, the United States Agency for International Development (USAID) and the World Health Organization (WHO) strongly recommended the ‘use of the digital revolution to scale up health interventions and engage civil society’ (World Health Organization, 2018). Patient engagement is a bedrock philosophy for healthcare policy and practice in the United States of America (USA) and the United Kingdom (UK) (Gibson et al., 2012; Millenson & Macri, 2012). Financial incentives and support are being offered for care systems that demonstrate practices of patient engagement in the USA and UK (Gibson et al., 2012; Millenson & Macri, 2012). Patient engagement integration into health policy has been discussed in the last decade; however, the operationalization, mention of a clear definition and monitoring of patient engagement as a structure, process and outcome lack consistency (van den Heuvel et al., 2018).

## 2 BACKGROUND

### 2.1 Principles of perinatal patient engagement

The principles of woman‐ and family‐centred care are central to perinatal care, consider the individual parent and aim for interactions between health providers and individuals that promote collaboration and shared decision‐making (Fontein‐Kuipers et al., 2018; Franck & O'Brien, 2019). Patient Engagement is conceptually linked to woman‐ and family‐centred care. The practices associated with patient engagement cannot be captured within a single measure or indicator (Barello et al., 2016; Higgins et al., 2017; Kelders, van Zyl, & Ludden, 2020). Higgins et al. (2017) proposed that the meaning of patient engagement deserved scrutiny, and other researchers pose that patient engagement is multifactorial and works through structures, processes and behaviours (Higgins et al., 2017; Kelders, Kip, & Greeff, 2020). In a concept analysis, patient engagement was defined as both a ‘process and behaviour [that] is shaped by the relationship between the patient and provider and the environment in which healthcare delivery takes place’ (Higgins et al., 2017). Four attributes of patient engagement provide conceptual components for inquiry: (1) access, (2) personalization, (3) commitment and (4) therapeutic alliance (Higgins et al., 2017). Access refers to the ability of the patient to obtain all health resources required to experience high‐quality and appropriate care (Higgins et al., 2017). Personalization assures that the interventions conform to the unique circumstances of the patient (Higgins et al., 2017). Commitment is the cognitive and emotional factors that empower the patient to exploit health resources and therapeutic alliance represents the elements of the patient–provider relationship that impact engagement in care (Higgins et al., 2017).

### 2.2 Examination of person‐centred perinatal eHealth practices

Perinatal eHealth programs have not been examined using clear definitions of person‐centred and patient engagement practices. Implementation of eHealth interventions in perinatal practice should begin with the definition of patient engagement and a clear understanding of person‐centred digital health interventions (DHI), as defined by the WHO (World Health Organization, 2018). WHO classifications of person‐centred DHI contain four categories of patient activities intended to support their health self‐management (World Health Organization, 2018). These four categories lay the foreground for person‐centred perinatal eHealth and are as follows: (1) Targeted client communication; (2) client‐to‐client communication; (3) personal health tracking and (4) on‐demand information services.

If integrating patient engagement into perinatal eHealth is to meet or exceed the promise as a novel system that supports current values of person‐centred perinatal practice, research needs to be conducted to examine the nature of perinatal eHealth, and how the attributes of patient engagement are being practised within programs. Here, this scoping review identifies the nature and range of person‐centred perinatal eHealth and illustrates how the attributes of patient engagement are practised within these programs. The research question guiding this review was: What is the nature and range of perinatal eHealth practice characterized by integration of the four WHO person‐centred DHI categories and patient engagement attributes?

## 3 METHOD

### 3.1 Design

A scoping review was suited for mapping person‐centred perinatal eHealth due to the complexity of this topic (Tricco et al., 2018). Considering the complexity and interdisciplinary nature of the perinatal eHealth practice we utilized an iterative process for data charting, analysis and synthesis recommended by Daudt et al. (2013) and endorsed by Pham et al. (2014). The aim of this scoping review was to develop an understanding of the nature and range of perinatal eHealth and identify gaps in the research to inform practice, policymaking and future research (Daudt et al., 2013). A systematic approach for this scoping review was further guided by the Preferred Reporting Items for Systematic Reviews and Meta‐Analyses—extension for scoping review (PRISMA‐ScR) guidelines (Tricco et al., 2018).

### 3.2 Search strategy

Five electronic databases (Web of Science, Scopus, PubMed, Eric and Cumulative Index to Nursing and Allied Health Literature) were searched in January 2020 and again on April 26th, 2022, to include all studies up to the end of 2021. We used an expansive list of search terms to incorporate person‐centred perinatal eHealth programs. See Table 1 for a list of general search terms and keywords. All citations were exported into RefWorks and Rayyan citation software for storage, screening and management (Ouzzani et al., 2016).

**TABLE 1:** Search keywords.

Keywords for search
Expectant Mothers, pregnancy, parent, family, partner, father AND Patient Engagement, Personalization, Decision Making, tailored care, tailoring information, tailoring resources, individual preferences, access to information, access to resources, access to guidance, healthcare availability, health service access, functional literacy, health literacy, commitment, patient commitment, motivation, patient‐provider relationship, therapeutic alliance, communication, empathy, mutual understanding, trust, therapeutic relationship AND Randomized Controlled Trial, group, feasibility, acceptability, exploratory, mixed‐method, Quasi‐Experimental Studies, non‐randomized controlled trial, qualitative studies AND Handheld, mobile, Computers, ipad, iphone, smartphone, cell phone, wireless, mHealth, Telemedicine, mobile health, eHealth, Wearable, application, External Fetal Monitoring, remote monitoring AND maternal care, antepartum, prenatal, perinatal, postnatal, neonatal, postpartum

### 3.3 Study selection

All reports included were published in English, had abstracts available, and no date limitations were set for the in original search. Studies were included that reported on person‐centred perinatal eHealth programs, target users were new or expectant parents, programs were delivered during pregnancy, 6–8 weeks after birth (puerperium) and in the case of neonatal care, from birth up to the time a neonate receives care in neonatal or public health services (commonly near 44 weeks postmenstrual age). Puerperium has been defined as 6 weeks after birth (Aisien, 2021); however, inconsistencies in reporting this period occur and often range from 6 to 8 weeks after birth. All programs would contain at least one of the four WHO patient‐centred DHI categories (World Health Organization, 2018). Studies were not included if the technology was meant to be used without a two‐way interaction between health providers and clients; the eHealth system was using only outdated forms of telehealth (i.e. follow‐up telehealth phone calls, paging or faxing) or the system was used solely for diagnostic screening.

### 3.4 Data charting

Descriptive characteristics of all included studies were charted by two researchers (J.A. & H.H.). Descriptive data included:

(1) Author, year and country, (2) Study design, (3) Aim, (4) Target population and setting, (5) Program structure/devices, (6) WHO DHI categories, (7) Engagement evaluation and (8) eHealth modalities. Deductive and inductive content were charted according to codebook.

### 3.5 Data analysis

Content analysis was performed for examining perinatal eHealth programs. Our initial codebook consisted of deductive codes related to access, personalization, commitment and therapeutic alliance and the four WHO DHI person‐centred categories (Higgins et al., 2017; Kyngäs et al., 2020a; World Health Organization, 2018). We ensured validity of our codebook development by separating maternity and neonatal studies, ensuring careful organization and separation of inductive meaning units that came from maternity and neonatal programs. eHealth modalities and perinatal treatments were inductively identified and defined through careful examination of data about the eHealth programs' structure and device use (Kyngäs et al., 2020b). Treatment and eHealth modality categories were added to the codebook after consultation with first, third and fourth authors (Kyngäs et al., 2020b). Next meaning units were identified based on a matrix of deductive and inductive concepts (Graneheim & Lundman, 2004). The first author extracted meaning units and suggested associated codes, these were reviewed by the last author for clarity and consistency of coding. Codes were developed from condensed meaning units from maternity and neonatal services separately (Graneheim & Lundman, 2004). Subcategories were developed from harmonization of codes, some codes in maternity and neonatal services overlapped and some remained unique (Graneheim & Lundman, 2004). The latent content of categories was formulated into two main themes (Graneheim & Lundman, 2004). Agreements about interpretations of the latent content were made in consultation between the first, second and fourth authors. The decisions stemming from these consultations support the fit of the evidence to the final interpretations of latent content (Tavory & Timmermans, 2014, pp. 105–106).

### 3.6 Ethics

This study did not require ethical approval or client consent.

## 4 RESULTS

### 4.1 Study selection

**FIGURE 1:**
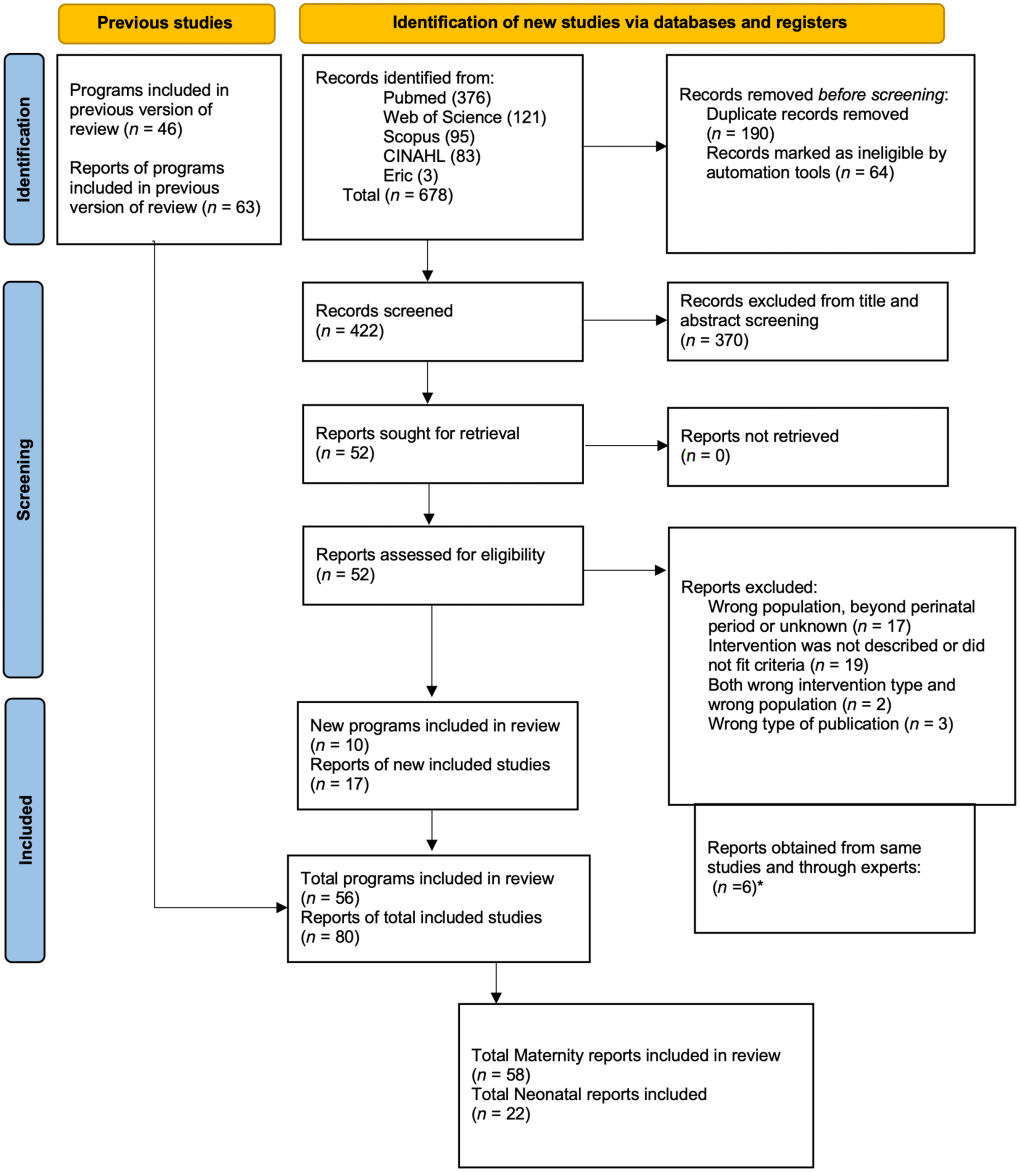
PRISMA 2020 flow diagram for updated systematic reviews.

First and third authors reviewed 1555 titles and abstracts independently. Full‐text review for screening was performed in 257 sources due to abstract inconsistency. The fourth author and a research assistant provided support when agreement was not reached, and 80 sources were selected for review (Maternity *n* = 58, Neonatal *n* = 22; See Figure 1; Page et al., 2021).

### 4.2 Study characteristics

**FIGURE 2:**
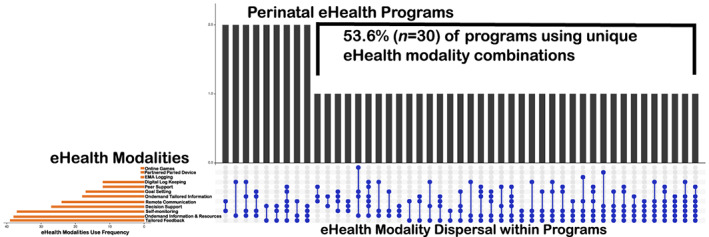
Perinatal eHealth programs and modalities.

**FIGURE 3:**
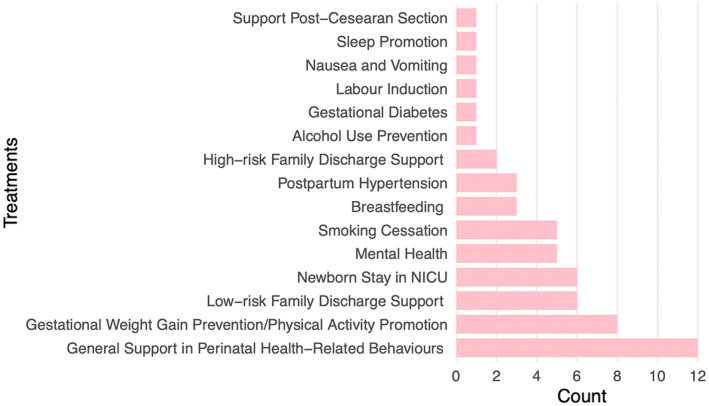
Treatments within Perinatal eHealth programs.

Thirty‐nine maternity programs and 17 neonatal programs were included in this review (*n* = 56). See Table S1 for charted data. Programs were delivered mainly in North America, the United Kingdom and Europe (See Table 2). Twelve eHealth modalities (See Figure 2) and 15 different treatments (Figure 3) were used in the programs. Programs integrated one to four of the WHO DHI person‐centred categories (Table S1). Healthcare providers included nurses, midwives, primary and special practice doctors, as well as public health, breastfeeding and co‐parenting experts, dieticians, lifestyle coaches and community health workers. In 53.6% (*n* = 30) of perinatal programs nurses were involved in provision of care, program development or research activities. All neonatal programs integrated nurses in provision of care, alternatively maternity programs reported nurses’ work in 16 out of 42 programs (38.1%). Fifteen percent of the studies were published in recognized nursing journals (Scimago Lab, 2021).

**TABLE 2:** Included studies' context and characteristics.

Study characteristics (*N* = 80)	Number of studies % (*N*)
Continent	
Asia	2.5 (2)
Australia/New Zealand	7.5 (6)
United Kingdom	18.7 (15)
Europe	17.5 (14)
North America	52.5 (42)
South America	1.2 (1)
Study design	
Qualitative Exploratory	18.75 (15)
User‐Centred/Design Research	13.75 (11)
Participatory Action Research/Implementation	10.0 (8)
Conference and other Reports of Development	7.5 (6)
Randomized control trial	50.0 (40)^a^
Nursing journal publications	
Maternity (*N* = 58)	10.3 (6)
Neonatal (*N* = 22)	27.3 (6)
Characteristics of eHealth programs (*N* = 56)	
Perinatal setting	
Maternity	75.0 (42)
Neonatal	25.0 (14)
Nurse involvement in perinatal eHealth	
Maternity *N* = 42	38.1 (16)
Neonatal *N* = 14	100.0 (14)

^a^ Eight of which were protocol reports; Two of which were mixed methods.

### 4.3 The nature of perinatal eHealth programs: Emergence of a complex structure of practice

Perinatal eHealth programs make up a structure of practice that developed through new interactions and processes mediated by eHealth modalities. The design and implementation of perinatal eHealth programs are emerging as the availability of new eHealth systems (i.e. applications and machine learning‐based tailored feedback), and ubiquitous devices (i.e. smartphones and wearables) increases. The current generation of new families identifies with perinatal eHealth (Danbjørg et al., 2015; Gund et al., 2013; Herring et al., 2019; Soltani et al., 2015). The modality combinations and use within programs are complex, used for a broad range of person‐centred care goals (See Figure 2). All programs are divided according to maternity or neonatal contexts (See Figure 3).

Programs for supporting parents at home in the care of their infants were found to be easy to use, relevant and understandable to users (Abbass‐Dick et al., 2017; Danbjørg et al., 2015). In one case, using an early discharge digital support for parents, a father was showing nurses how to use the technology and his partner stated, ‘my boyfriend is technical, so it was [basically] him showing the nurses how it worked’ (Danbjørg et al., 2014). Pregnant women expressed comfort in using devices and applications that they could take with them anywhere, to receive information anytime (Himes et al., 2017; Wierckx et al., 2014). Users expressed wanting to use the programs beyond the study periods and wished for more harmonized systems throughout the entire perinatal period (Krishnamurti et al., 2017; Shorey et al., 2018). Most programs across both maternity and neonatal contexts were focused on a single care objective.

### 4.4 Practising patient engagement within perinatal eHealth

Unique practices related to each attribute of patient engagement are summarized in Table 3 and described below in a more detailed narrative synthesis. Access and personalization were integrated into each program, whereas commitment and therapeutic alliance were absent from 3 and 8 programs respectively (See Supplementary Material S4: Table S3).

**TABLE 3:** Novel practices of patient engagement within eHealth: Categories and sub‐categories from content analysis.

Patient engagement categories	Subcategories of practices of patient engagement within eHealth % (*N*) of programs			
**Access** Receival of resources, guidance and tools. Adaptability of services for personalized access.^a^	Maternity 100 (39)	Access has the potential to support increasing health knowledge, skill and management of care needs with new eHealth modalities **56.4 (23)** Access supports new opportunities for participation **94.9 (37)** Access to eHealth allows for receiving appropriate care through new care interactions **89.7 (35)**	Neonatal 100 (17)	Access gives opportunities for support for increasing health knowledge, skill and confidence building with new eHealth modalities **70.6 (12)** Parents get to practise new ways of caring for self and newborns by having access to eHealth **70.6 (12)** Access to eHealth program modalities allows for more opportunities for appropriate care **41.2 (7)**
**Personalization** Provision of unique and tailored services that align with clients' life circumstances and diversity.^a^	Maternity 100 (39)	Designed systems consider the diversity and preferences of target users **61.5 (24)** The individual perinatal eHealth user is considered throughout healthcare journey **43.6 (17)** Perinatal eHealth users receive a personal care experience **43.6 (17)**	Neonatal 100 (17)	Designed systems consider the diversity of target users **76.5 (13)** The individual family is considered throughout healthcare journey **58.8 (10)** Parents experience personal eHealth care interactions **58.8 (10)**
**Commitment** Harnessing cognitive and emotional factors that empower clients to exploit health resources available. Commitment is demonstrated by client efforts over time and is more inclusive than simple motivation that may waiver according to changing circumstances.^a^	Maternity 94.9 (37)	Commitment examined through behaviour theories of change **56.4 (22)** New ways of becoming committed were stimulated through new eHealth care interactions **59.0 (23)** eHealth supported coaching and connectivity in new ways **74.4 (29)** Meaningful involvement is a demonstration of commitment and was enabled in new ways with the use of digital health **56.4 (22)**	Neonatal 94.1 (16)	Commitment examined through behaviour and self‐efficacy theories **5.9 (1)** New ways of Becoming committed were stimulated through new eHealth care interactions **52.9 (9)** eHealth supported coaching and connectivity in new ways **58.8 (10)** eHealth mediates new opportunities for parents to become meaningfully involved in the care processes of their infants **17.6 (3)**
**Therapeutic Alliance** Incorporates elements of the client‐provider relationship including quality of the clinical interaction, communication, empathy, or mutual understanding.^a^	Maternity 82.1 (32)	Professional caregivers and patients interact in new ways with the use of eHealth modalities **46.2 (18)** Some eHealth functionalities are seen as ‘partners in care’ **76.5 (13)** Emotional and lifesaving support can be received using eHealth modalities **58.8 (10)** Integration of eHealth modalities into current practice structure has potential to support sustainability of programs (ensure capacity for eHealth practice is supported) **41.0 (16)** Perinatal eHealth users want to have communication and perform teamwork with professional caregivers using eHealth modalities, but this was not used in the program due to objections from providers **2.6 (1)**	Neonatal 94.1 (16)	Professional caregivers and patients interact in new ways with the use of eHealth modalities **64.7 (11)** Some eHealth functionalities are seen as ‘partners in care’ **29.4 (5)** Emotional and lifesaving support can be received using eHealth modalities **23.5 (4)** Integration of eHealth modalities into current practice structure has potential to support sustainability of programs (ensure capacity for eHealth practice is supported; involve the perspectives for sustainability from care staff to ensure possibility for ‘buy in’) **64.7 (11)** Perinatal eHealth users want to have communication and perform teamwork with professional caregivers using eHealth modalities, but this was not used in the program due to objections from providers **5.9 (1)**

^a^ Higgins, T., Larson, E., & Schnall, R. (2017). Unraveling the meaning of patient engagement: A concept analysis. *Patient Education and Counseling*, *100*(1), 30–36. https://doi.org/10.1016/j.pec.2016.09.002.

#### 4.4.1 Access

Access is practised in programs through the provision of eHealth modalities that support new opportunities for new or expectant parents to participate in self‐care, health promotion and illness prevention. eHealth modalities mediate new interactions that support the access to appropriate care and potentiate support for increased knowledge, skill and capacity for self‐management of pregnant persons' and families' wellness and development.

eHealth programs aimed to give access to on‐demand health information and resources, communication and tailored feedback intended to support families in building confidence, familiarity, knowledge and awareness in health promotion and illness prevention activities (Banerjee et al., 2020; Baron et al., 2018; Cramer et al., 2018; Doherty et al., 2019; Fontein‐Kuipers et al., 2016; Shorey et al., 2018; Spargo & Vries, 2018; Strand et al., 2021; Wierckx et al., 2014). Patients had more convenient communication experiences with their health professionals, timely information through feedback and self‐monitoring modalities, and could lead content and timing of communication (Dalton et al., 2018; Doherty et al., 2019; Herring et al., 2019; Holm et al., 2019). Medical and non‐medical issues were brought to the forefront of the maternity patients' minds through access to information and communication (Carrilho et al., 2019; de Mooij et al., 2018; Himes et al., 2017; Krishnamurti et al., 2017; O'Brien et al., 2013; Soltani et al., 2015). Parents had opportunities to be involved in the care of their infants in new ways through access to NICU automated updates through short message service (SMS) (Globus et al., 2016), and infant care and collaboration training using education and coaching applications (Banerjee et al., 2020; Platonos et al., 2018). Women and their families received new access to the care team from remote locations which supported timely appropriate care, in many cases from the comfort of their own homes (Doherty et al., 2020; Garne et al., 2016; Gund et al., 2013; Holm et al., 2019; Payakachat et al., 2020; Shorey et al., 2018; Strand et al., 2021; Triebwasser et al., 2020).

#### 4.4.2 Personalization

User‐centred program design practices supported personalization. eHealth practices included the provision of personal care experiences that were founded on woman‐and family‐centred approaches. Personalization practices encompass tailored, on‐demand, flexible programs and consider new and expectant parents' preferences, needs, values and diversity.

Programs were very often designed using a participatory design (PD), or user‐centred design model. Involving key stakeholders (e.g. professional, informal caregivers and patients) in the design of eHealth programs was seen to encourage engagement and sustainable uptake of perinatal programs (Danbjørg et al., 2015; Payakachat et al., 2020; Strand et al., 2021). One research team in Canada developed a way of recording interactions with the C‐Care application throughout real‐time testing and modified the program during testing to accommodate higher interaction with the system (Ke et al., 2021). Functions included automated text messages personalized to the individual's unique circumstances, which supported core woman‐and family‐centred concepts such as reciprocity, tailored care and shared decision‐making (Danbjørg et al., 2015; Doherty et al., 2020). Users expressed that having understandable, individualized, relevant and timely information met their support needs during pregnancy, labouring at home and in early days at home with their infants (de Mooij et al., 2018; Frize et al., 2013; Gibson et al., 2021; O'Brien et al., 2013; Ridgeway et al., 2015; Willcox et al., 2015; Yee et al., 2021). One woman described that she felt a personal care experience while using remote foetal monitoring system at home, because nurses could see what was happening on their own monitors and coordinate with her at a distance (O'Brien et al., 2013). While parents using an Application to support early discharge home after birth found that staying home and getting timely answers to their questions using remote communication had a positive impact on their affinity within the family (Danbjørg et al., 2015).

#### 4.4.3 Commitment

Commitment is practised in programs through integration of behavioural change and self‐efficacy theories during the development and design (See Supplement Material S3: Table S2). eHealth modalities mediated new opportunities for patients to become meaningfully involved in their own care processes. Commitment was also practised through the supporting of new ways to coach and connect with patients. Lastly, new interactions mediated by eHealth modalities supported new processes of becoming committed to self and newborn care.

Perinatal eHealth programs change the face of connectivity and coaching for new or expectant parents. Tailored alerts and information sharing directed to the personal handheld devices of patients changes their capacity to interact as members of the care teams (Choi et al., 2015; Danbjørg et al., 2015; Davis et al., 2018; Frize et al., 2013; Herring et al., 2019). eHealth patients have a chance to view information and their own personal health data and trends on demand (Abbass‐Dick et al., 2017; Isetta et al., 2013; Valencia et al., 2020; van der Wulp et al., 2014). These opportunities are mediated by eHealth modalities such as tailored feedback, decision‐making supports, digital log keeping and self‐monitoring. Perinatal patients can participate in shared decision‐making with new confidence and receive contact and coaching when and where they would like it (Danbjørg et al., 2015; de Mooij et al., 2018). Meaningful involvement in perinatal care processes was a motivating factor for many perinatal patients. They could look at their own personal trends, record and report their findings (i.e. for newborn assessment or pregnancy weight gain or blood pressures) and support care decisions and goal setting with their professional care givers (Davis et al., 2018; Dougall et al., 2020; Garfield et al., 2016; Isetta et al., 2013; Rhoads et al., 2017).

Perinatal patients experienced becoming committed for self and newborn care enabled through new interactions mediated by eHealth modalities. Maternity programs supported motivation for behaviour changes through interactive tools, and feedback, as was seen in Doherty and colleagues ‘ideas machine’ a feedback system that used user input about preferences and experiences to deliver tailored tips for achieving goals for mental wellness in the moment (Doherty et al., 2019). Parents of newborns were able to become committed to learning and practising their new roles when just returning from hospital with access to on‐demand information and resources that were provided in many formats, instructional videos, links to go deeper on topics, and pages they could ‘favourite’ for reading later (Danbjørg et al., 2015; Garfield et al., 2016; Isetta et al., 2013; Shorey et al., 2018; Strand et al., 2021).

#### 4.4.4 Therapeutic alliance

Perinatal eHealth practices that integrate therapeutic alliance encompass new provider–patient interactions, and eHealth‐driven emotional and lifesaving supportive activities. eHealth components are seen as partners in care, and the fostering of teamwork through remote communication are important features from the patients' perspective. Therapeutic alliance is supported through consideration for the integration of eHealth modalities into workflows, and eHealth policies.

Therapeutic alliance is practised through considering policy and physical infrastructure and staff attitudes, and capacity to use eHealth modalities as guiding factors for successful implementation of new eHealth practices (Banerjee et al., 2020; Baruth et al., 2019; Bower et al., 2005; Dalton et al., 2018; Danbjørg et al., 2015; Doherty et al., 2020; Frize et al., 2013; Globus et al., 2016; Herring et al., 2019; Jefferson et al., 2019; Strand et al., 2021; Triebwasser et al., 2020; Wierckx et al., 2014). Self‐monitoring and sharing of data to aid in collaborative clinical interpretations and decision‐making emerged as new patient–provider interactive processes. Some perinatal patients used data to guide conversations, and other times clinicians were triggered through the automated systems to contact patients because of concerning data or events (i.e. abnormal blood pressure readings or mental health alerts) (Hantsoo et al., 2018; Krishnamurti et al., 2017; O'Brien et al., 2013; Rhoads et al., 2017; Strand et al., 2021). Self‐monitoring and remote communication modalities mediated lifesaving and emotional support provision by professional caregivers at a distance (Doherty et al., 2020; Holm et al., 2019; Jefferson et al., 2019; Ledford et al., 2017; Marko et al., 2016; Rhoads et al., 2017; Strand et al., 2021). Perinatal eHealth patients explained that they felt companionship with some eHealth components, with one women saying that the eHealth program was the ‘only person in [her] life who asked…how [she] was doing everyday’ (Krishnamurti et al., 2017). Interactions between perinatal patients and the eHealth modalities provided new forms of support to supplement face‐to‐face visits (Banerjee et al., 2020; Danbjørg et al., 2015; Doherty et al., 2020; Herbec et al., 2014; Himes et al., 2017; Hirshberg et al., 2018; Holm et al., 2019; Ledford et al., 2017; Shorey et al., 2018; Soltani et al., 2015; Strand et al., 2021; van der Wulp et al., 2014; Yee et al., 2021).

## 5 DISCUSSION

### 5.1 Principal results

This is the first review to bring together perinatal eHealth programs, treatments and modalities, with the aim of describing the range of practices and conceptualizing the nature of perinatal person‐centred eHealth. Perinatal eHealth programs in the developed world make up a structure of practice that contains person‐centred eHealth modalities and separates care between maternity and neonatal practices. Nursing leadership might be lacking in the structure development and process evaluation of perinatal eHealth due to the high percentage of studies and programs that are not reporting on nursing expertise. Access and personalization are being practised in all perinatal eHealth programs, and commitment and therapeutic alliance are lacking in a 19.6% (*n* = 11) of all programs. Findings from this review reveal that person‐centred and patient engagement practices are being used within the current structure; however, development and design of these programs lack harmonization between maternity and neonatal care, and consistency of commitment and therapeutic alliance practices.

### 5.2 The nature of perinatal eHealth programs

A summary of the programs captured in this scoping review illustrates that perinatal eHealth is being provided across various perinatal treatments; from health promotion and symptom management in pregnancy, to parental skill development in caring for, breastfeeding and monitoring infants and supporting parental‐infant closeness (See Figure 3). No programs have harmonized maternity and neonatal treatments across the continuum of the perinatal period into a single eHealth program. A structure of siloed care has been persistent in perinatal care programs internationally due to the growing complexity and specialization of services (Liu, 2016; Molenaar et al., 2020). Uncoordinated services have led to low engagement by families (Molenaar et al., 2018). Research has revealed that new and expectant parents desire an expanded integrated service that supports easy navigation and a smoother continuity of care throughout their perinatal journeys (Abbass‐Dick et al., 2017; Danbjørg et al., 2015; Garne Holm et al., 2017; Himes et al., 2017; Liu, 2016; Wierckx et al., 2014). Our findings reveal that although eHealth programs could provide a system for harmonizing maternity and neonatal care programs this potential has not yet been harnessed.

A lot has been learned about how to integrate numerous eHealth modalities into routine and common perinatal care processes (i.e. management and monitoring of gestational diabetes and hypertension; and supporting parent participation in the care of a sick neonate). Programs included in this review have innovated clinical care practices to include eHealth modalities with the aim of improving patient satisfaction, health and clinical outcomes. The WHO recommends clearly articulating how technology will address specific person‐centred health system problems, such as poor patient experience and delayed provision of care (World Health Organization, 2018). Therefore, the WHO person‐centred digital health interventions being implemented by each perinatal eHealth program in this review could be more clearly identified by researchers in the future to support better understanding of the usefulness of eHealth innovation towards solving person‐centred health system challenges. In combination with this nursing‐led research about perinatal eHealth practice and program development should be considered. Exemplary nursing leadership has been found to positively impact on structural outcomes for quality care, supports common visions and goals for care among staff and promotes effective information sharing (Cook & Leathard, 2004; Kiwanuka et al., 2021; Sfantou et al., 2017).

### 5.3 Practising patient engagement within perinatal eHealth

#### 5.3.1 Access

Access has been identified as a precondition for patient engagement (Kelders, van Zyl, & Ludden, 2020) and as a metric that should be considered when examining the presence of patient engagement within eHealth programs (Barello et al., 2016). Our review expands on this by illustrating that practices of access provide opportunities for developing partnerships at a distance and allow for new participation in perinatal care processes. Pregnant persons and families can integrate perinatal practices into their daily lives. New access can lead to care approaches that connect providers with patients in their natural settings. This has provided relief to parents who find it hard to make the trips to medical offices, and balances power dynamics as providers are assessing families in their own home environments through video conferencing (Lieu et al., 2021). Pregnant persons monitor their own goals for health‐related behaviours without waiting to have important assessments and collation of lifestyle pattern data during antenatal clinic visits (Naughton et al., 2013; van der Wulp et al., 2014). Research about self‐monitoring has suggested that self‐care activities might introduce increased burden related to worry and stress (Auxier et al., 2023; Mol, 2018, p.19). Further study should be conducted on the nature of care processes occurring at home from a variety of perspectives and user groups. Perinatal eHealth practitioners should also consider tailoring the level of access provided and the amount of engagement that suites each client when using eHealth programs.

#### 5.3.2 Personalization

In this review, eHealth modalities were mechanized for personalization practices, and user‐centred design of programs contributed to the integration of personalization from a development perspective. Past literature shows that perinatal services do not always support women and families' expectations for personalized care (Auxier, 2017; Platonos et al., 2018). This scoping review reveals that eHealth modalities mediate new personal care experiences. By using eHealth modalities purposefully for the sustainment of person‐centred care, and the tailoring of care journeys to unique patients some of the persistent challenges with enabling person‐centred care might be combated. Patient involvement was common in programs from this review and in line with the best practice recommendation of ensuring stakeholder involvement in eHealth program design (Oberschmidt et al., 2022).

#### 5.3.3 Commitment

Our findings reveal that consistency in the use of process measures to guide evaluation of commitment and participation within perinatal eHealth programs is lacking. Process evaluation, also described as process monitoring by the WHO is needed for collecting and analysing data to understand how well our programs are meeting the aims of care (World Health Organization, 2016). Commitment can be measured through behaviour and cognition, as seen in Kelders, Kip, and Greeff (2020) measure, Twente Engagement with Ehealth Technologies Scale (TWEETS). Neonatal eHealth person‐centred practices that support commitment can be evaluated by using a newly developed process evaluation measure, the CO‐PARTNER tool (van Veenendaal et al., 2021). More process measures could be developed in the future to guide the monitoring of perinatal eHealth user engagement and care processes related to commitment as these are not being consistently reported in the scientific literature. This scoping review highlights the potential to monitor behavioural engagement and participation over time using digital log keeping and ecological momentary assessment modalities.

#### 5.3.4 Therapeutic alliance

Therapeutic alliance sets patient engagement as a concept apart from others such as empowerment, and involvement (Higgins et al., 2017). While there has been a plethora of knowledge accumulated about collaboration and connectedness between perinatal care providers and their clients, very little is known about how therapeutic alliance is enacted within perinatal eHealth programs. Our findings illustrated that in 12.5% of programs therapeutic alliance practices were not reported. Current research shows that increased connectivity can aid in collaboration and continuity of perinatal care and our review highlights which functionalities help to enact these practices. More purposeful inquiry into this attribute of patient engagement would support deeper understandings of the nuanced interactions between patients, providers and eHealth modalities. All care begins with building trust, this is being investigated in relation to face‐to‐face perinatal practice (Korstjens, 2021; Wreesmann et al., 2021); however, researchers and clinicians need to appreciate the importance of investigating how trust is built with eHealth systems as a partner‐in‐care. Person‐centred eHealth modalities are helping to bring relevant, personal and timely resources, information, and support to perinatal clients and help to provide safer transition from hospital to home. In the wake of a revolution in perinatal practice, providers need to be supported to interact with eHealth systems in ways that enhance and support the co‐creation of therapeutic alliances.

### 5.4 Implications for nursing research and practice

This review demonstrates a synthesis of knowledge from many disciplines. From this, we have a diversity of perspectives that provides a shared understanding of the range and nature of perinatal eHealth. However, nursing inquiry and practice are scarce in the literature related to maternity eHealth practice, neonatal literature has integrated nursing expertise and inquiry to a larger extent. Although multidisciplinary work is of high importance, nursing knowledge and inquiry are lacking in the research and development of services overall. Health Science literature indicates that nursing and midwifery inquiry is integral to the development, implementation and evaluation of eHealth resource use in perinatal services (Richardson et al., 2018). More collaborative research should be conducted that combines user design theory with nursing science perspectives.

Findings from our review illustrate that eHealth modalities support women and families towards accessible, and personalized health service, eHealth modalities should be paired with relational nursing approaches (Korstjens, 2021; Stelwagen et al., 2020). Commitment and therapeutic alliance integration within perinatal eHealth fulfils perinatal nursing practice goals of woman‐ and family‐centred care; parent–infant closeness and health‐related behaviour promotion in pregnancy (Fontein‐Kuipers et al., 2018; Franck & O'Brien, 2019; van den Heuvel et al., 2018). In this review, we recommend prioritizing defining and implementing commitment and therapeutic alliance interventions within perinatal eHealth as this will support more clarity for nursing practitioners working towards evidence‐based practices (EBP).

### 5.5 Limitations and strengths

While this scoping review provides a new entry point in which to discuss and appreciate perinatal eHealth, the nature of terminology usage in the available publications is inconsistent and we suspect some sources have been missed due to the complexity and interdisciplinary nature of the literature. Key terms were not used to capture pregnancy experiences of person's not identifying as women, (i.e. trans, trans/masculine and non‐binary and transgender). Future reviews discussing perinatal care should include this group, to better identify the level of their involvement in perinatal eHealth evaluation. Further, this work is limited in its form as a scoping review and the level of evidence cannot be evaluated as such. We attended to credibility through careful consideration of suitable meaning units that were based on definitions of patient engagement attributes and WHO digital service person‐centred categories (Graneheim & Lundman, 2004). Transferability can be judged through our clear descriptions of the practice structure context and presentation of findings (Graneheim & Lundman, 2004). We suggest avenues for perinatal eHealth implementation, clinical practice and policy considerations and future research based on descriptions of the nature and range of perinatal eHealth and current knowledge gaps.

## 6 CONCLUSIONS

Perinatal eHealth is emerging as a complex and potentially harmonized practice, the next generations of new families demand access to personalized, relevant, stimulating, integrated and connected perinatal care. To date, current evaluations of perinatal eHealth programs have been mainly focused on satisfaction of care, feasibility and medical‐based patient outcomes. Process evaluation and purposeful eHealth program development should be carried out more commonly in the future and can incorporate more nursing perspectives. Based on the findings from this review, access and personalization are being practised in all included programs, but therapeutic alliance and commitment can be reported more often. The integration of all attributes is important for embedding core values of person‐centred perinatal care into practice. The next steps stemming from this review are to conduct an interpretive synthesis to inform a patient engagement model for perinatal eHealth development and quality assurance.

## CONFLICT OF INTEREST STATEMENT

We have no conflicts of interest to disclose.

## Supporting information


Data S1.

## Data Availability

The data that supports the findings of this study are available in the supplementary material of this article.

## References

[nop21822-bib-0001] Abbass‐Dick, J. , Xie, F. , Koroluk, J. , Alcock Brillinger, S. , Huizinga, J. , Newport, A. , Goodman, W. M. , & Dennis, C. L. (2017). The development and piloting of an eHealth breastfeeding resource targeting fathers and partners as co‐parents. Midwifery, 50, 139–147. 10.1016/j.midw.2017.04.004 28448858

[nop21822-bib-0002] Aisien . (2021). The puerperium. In contemporary obstetrics and gynecology for developing countries. Springer, Champions.

[nop21822-bib-0101] Auxier, J. , Savolainen, K. T. , Bender, M. , Rahmani, A. M. , Sarhaddi, F. , Azimi, I. , & Axelin, A. M. (2023). Exploring access as a process of adaptation in a self‐monitoring perinatal ehealth system: Mixed methods study from a sociomaterial perspective. JMIR Formative Research, 7, e44385. 37184929 10.2196/44385 PMC10227706

[nop21822-bib-0004] Auxier, J. N. (2017). The influence of environments on fear of childbirth during women's intrapartum hospital stays. (Master's Thesis). The University of British Columbia. cIRcle repository.

[nop21822-bib-0102] Banerjee, J. , Aloysius, A. , Mitchell, K. , Silva, I. , Rallis, D. , Godambe, S. V. , & Deierl, A. (2020). Improving infant outcomes through implementation of a family integrated care bundle including a parent supporting mobile application. Archives of Disease in Childhood ‐ Fetal and Neonatal Edition, 105(2), 172–177. 10.1136/archdischild-2018-316435 31227521

[nop21822-bib-0005] Barello, S. , Triberti, S. , Graffigna, G. , Libreri, C. , Serino, S. , Hibbard, J. , & Riva, G. (2016). eHealth for patient engagement: A systematic review. Frontiers in Psychology, 6, 2013. 10.3389/fpsyg.2015.02013 26779108 PMC4705444

[nop21822-bib-0103] Baron, A. M. , Ridgeway, J. L. , Finnie, D. M. , Stirn, S. L. , Morris, M. A. , Branda, M. E. , Inselman, J. W. , & Baker, C. A. (2018). Increasing the connectivity and autonomy of RNs with low‐risk obstetric patients: Findings of a study exploring the use of a new prenatal care model. American Journal of Nursing, 118(1), 48–55. 10.1097/01.NAJ.0000529715.93343.b0 29280806

[nop21822-bib-0104] Baruth, M. , Schlaff, R. A. , Deere, S. , Walker, J. L. , Dressler, B. L. , Wagner, S. F. , Boggs, A. , & Simon, H. A. (2019). The feasibility and efficacy of a behavioral intervention to promote appropriate gestational weight gain. Maternal & Child Health Journal, 23(12), 1604–1612. 10.1007/s10995-019-02812-6 31541375

[nop21822-bib-0105] Bower, D. J. , Barry, N. , Reid, M. , & Norrie, J. (2005). Designing and implementing E‐health applications in the UK's National Health Service. Journal of Health Communication, 10(8), 733–750. 10.1080/10810730500326732 16316936

[nop21822-bib-0106] Carrilho, J. M. , Oliveira, I. J. R. , Santos, D. , Osanan, G. C. , Cruz‐Correia, R. J. , & Reis, Z. S. N. (2019). Pregnant users’ perceptions of the birth plan interface in the “My Prenatal Care” app: Observational validation Study. JMIR Formative Research, 3(1), e11374. 10.2196/11374 30920372 PMC6458531

[nop21822-bib-0107] Choi, J. , Lee, J. H. , Vittinghoff, E. , & Fukuoka, Y. (2015). mHealth physical activity intervention: A randomized pilot study in physically inactive pregnant women. Maternal and Child Health Journal, 20(5), 1091–1101. 10.1007/s10995-015-1895-7 PMC4826820 26649879

[nop21822-bib-0006] Cook, M. J. , & Leathard, H. L. (2004). Learning for clinical leadership. Journal of Nursing Management, 12(6), 436–444. 10.1111/j.1365-2834.2004.00420.x 15509273

[nop21822-bib-0108] Cramer, M. E. , Mollard, E. K. , Ford, A. L. , Kupzyk, K. A. , & Wilson, F. A. (2018). The feasibility and promise of mobile technology with community health worker reinforcement to reduce rural preterm birth. Public Health Nursing, 35(6), 508–516. 10.1111/phn.12543 30216526

[nop21822-bib-0109] Dalton, J. A. , Rodger, D. , Wilmore, M. , Humphreys, S. , Skuse, A. , Roberts, C. T. , & Clifton, V. L. (2018). The Health‐e Babies App for antenatal education: Feasibility for socially disadvantaged women. PLoS One, 13(5), e0194337. 10.1371/journal.pone.0194337 29768407 PMC5955503

[nop21822-bib-0110] Danbjørg, D. B. , Wagner, L. , & Clemensen, J. (2014). Designing, developing, and testing an app for parents being discharged early postnatally. Journal for Nurse Practitioners, 10(10), 794–802. 10.1016/j.nurpra.2014.07.023

[nop21822-bib-0111] Danbjørg, D. B. , Wagner, L. , Kristensen, B. R. , & Clemensen, J. (2015). Intervention among new parents followed up by an interview study exploring their experiences of telemedicine after early postnatal discharge. Midwifery, 31(6), 574–581. 10.1016/j.midw.2015.02 25765743

[nop21822-bib-0112] Davis, D. , Davey, R. , Williams, L. T. , Foureur, M. , Nohr, E. , Knight‐Agarwal, C. , Lawlis, T. , Oats, J. , Skouteris, H. , & Fuller‐Tyszkiewicz, M. (2018). Optimizing gestational weight gain with the Eating4two Smartphone App: Protocol for a randomized controlled trial. JMIR Research Protocols, 7(5), e146. 10.2196/resprot.9920 29848468 PMC6000478

[nop21822-bib-0007] Daudt, H. M. , van Mossel, C. , & Scott, S. J. (2013). Enhancing the scoping study methodology: A large, inter‐professional team's experience with Arksey and O'Malley's framework. BMC Medical Research Methodology, 13(1), 48. 10.1186/1471-2288-13-48 23522333 PMC3614526

[nop21822-bib-0113] de Mooij, M. J. M. , Hodny, R. L. , O’Neil, D. A. , Gardner, M. R. , Beaver, M. , Brown, A. T. , Barry, B. A. , Ross, L. M. , Jasik, A. J. , Nesbitt, K. M. , Sobolewski, S. M. , Skinner, S. M. , Chaudhry, R. , Brost, B. C. , Gostout, B. S. , & Harms, R. W. (2018). OB nest: Reimagining low‐risk prenatal care. Mayo Clinic Proceedings, 93(4), 458–466. 10.1016/j.mayocp.2018.01 29545005

[nop21822-bib-0114] Doherty, K. , Barry, M. , Belisario, J. M. , Morrison, C. , Car, J. , & Doherty, G. (2020). Personal information and public health: Design tensions in sharing and monitoring wellbeing in pregnancy. International Journal of Human‐Computer Studies, 135, 102373. 10.1016/j.ijhcs.2019.102373 32127731 PMC6959837

[nop21822-bib-0115] Doherty, K. , Marcano‐Belisario, J. , Cohn, M. , Mastellos, N. , Morrison, C. , Car, J. , & Doherty, G. (2019). Engagement with mental health screening on mobile devices: results from an antenatal feasibility study . Proceedings of the 2019 CHI Conference on Human Factors in Computing Systems, 1–15 10.1145/3290605.3300416

[nop21822-bib-0116] Dougall, G. , Franssen, M. , Tucker, K. L. , Yu, L.‐M. , Hinton, L. , Rivero‐Arias, O. , Abel, L. , Allen, J. , Band, R. J. , Chisholm, A. , Crawford, C. , Green, M. , Greenfield, S. , Hodgkinson, J. , Leeson, P. , McCourt, C. , MacKillop, L. , Nickless, A. , Sandall, J. , … McManus, R. J. (2020). Blood pressure monitoring in high‐riskpregnancy to improve the detection and monitoring of hypertension (the BUMP 1 and 2 trials): Protocol for two linked randomised controlled trials. BMJ Open, 10(1), e034593. 10.1136/bmjopen-2019-034593 PMC7044851 31980512

[nop21822-bib-0117] Fontein‐Kuipers, J. A. C. A. , & de Vries, R. G. (2016). WazzUp mama? The development of an intervention to prevent and reduce maternal distress during pregnancy. Archives of Women's Mental Health, 19(5), 779–788. 10.1007/s00737-016-0614-8 26965708

[nop21822-bib-0008] Fontein‐Kuipers, Y. , de Groot, R. , & van Staa, A. (2018). Woman‐centered care 2.0: Bringing the concept into focus. European journal of Midwifery, 2(5), 1–12. 10.18332/ejm/91492 PMC7846029 33537566

[nop21822-bib-0009] Franck, L. S. , & O'Brien, K. (2019). The evolution of family‐centered care: From supporting parent‐delivered interventions to a model of family integrated care. Birth Defects Research, 111(15), 1044–1059. 10.1002/bdr2.1521 31115181

[nop21822-bib-0118] Frize, M. , Bariciak, E. , & Gilchrist, J. (2013). PPADS: Physician‐PArent Decision‐Support for neonatal intensive care. In C. U. Lehmann , E. Ammenwerth , & C. Nøhr (Eds.), MEDINFO 2013 (pp. 23–27). IOS Press. 10.3233/978-1-61499-289-9-23 23920508

[nop21822-bib-0119] Garfield, C. F. , Lee, Y. S. , Kim, H. N. , Rutsohn, J. , Kahn, J. Y. , Mustanski, B. , & Mohr, D. C. (2016). Supporting parents of premature infants transitioning from the NICU to home: A pilot randomized control trial of a smartphone application. Internet Interventions, 4, 131–137. 10.1016/j.invent.2016.05.004 27990350 PMC5156477

[nop21822-bib-0121] Garne, K. , Brodsgaard, A. , Zachariassen, G. , & Clemensen, J. (2016). Telemedicine in neonatal home care: Identifying parental needs through participatory design. JMIR Research Protocols, 5(3), 274–281. 10.2196/resprot.5467 PMC4958140 27392576

[nop21822-bib-0120] Garne Holm, K. , Brødsgaard, A. , Zachariassen, G. , Smith, A. C. , & Clemensen, J. (2017). Participatory design methods for the development of a clinical telehealth service for neonatal homecare. SAGE Open Medicine, 5, 205031211773125. 10.1177/2050312117731252 PMC5613838 28975028

[nop21822-bib-0010] Gibson, A. , Britten, N. , & Lynch, J. (2012). Theoretical directions for an emancipatory concept of patient and public involvement. Health: An Interdisciplinary Journal for the Social Study of Health, Illness and Medicine, 16(5), 531–547. 10.1177/1363459312438563 22535648

[nop21822-bib-0122] Gibson, C. , Ross, K. , Williams, M. , & de Vries, N. (2021). The experiences of mothers in a neonatal unit and their use of the babble app. SAGE Open, 11(2), 215824402110231. 10.1177/21582440211023170

[nop21822-bib-0123] Globus, O. , Leibovitch, L. , Maayan‐Metzger, A. , Schushan‐Eisen, I. , Morag, I. , Mazkereth, R. , Glasser, S. , Kaplan, G. , & Strauss, T. (2016). The use of short message services (SMS) to provide medical updating to parents in the NICU. Journal of Perinatology, 36(9), 739–743. 10.1038/jp.2016.83 27195981

[nop21822-bib-0124] Graneheim, U. H. , & Lundman, B. (2004). Qualitative content analysis in nursing research: Concepts, procedures and measures to achieve trustworthiness. Nurse Education Today, 24(2), 105–112. 10.1016/j.nedt.2003.10.001 14769454

[nop21822-bib-0125] Gund, A. , Sjöqvist, B. A. , Wigert, H. , Hentz, E. , Lindecrantz, K. , & Bry, K. (2013). A randomized controlled study about the use of eHealth in the home health care of premature infants. BMC Medical Informatics and Decision Making, 13(1), 22. 10.1186/1472-6947-13-22 23394465 PMC3583709

[nop21822-bib-0126] Hantsoo, L. , Criniti, S. , Khan, A. , Moseley, M. , Kincler, N. , Faherty, L. J. , Epperson, C. N. , & Bennett, I. M. (2018). A mobile application for monitoring and management of depressed mood in a vulnerable pregnant population. Psychiatric Services, 69(1), 104–107. 10.1176/appi.ps.201600582 29032705 PMC5750085

[nop21822-bib-0127] Hawkins, M. , Iradukunda, F. , & Paterno, M. (2019). Feasibility of a sleep self‐management intervention in pregnancy using a personalized health monitoring device: Protocol for a pilot randomized controlled trial. JMIR Research Protocols, 8(5), 118–128. 10.2196/12455 PMC6658274 31144670

[nop21822-bib-0011] He, F. B. , Axelin, A. , Ahlqvist‐Björkroth, S. , Raiskila, S. , Löyttyniemi, E. , & Lehtonen, L. (2021). Effectiveness of the close collaboration with parents intervention on parent‐infant closeness in NICU. BMC Pediatrics, 21(1), 28. 10.1186/s12887-020-02474-2 33430816 PMC7798198

[nop21822-bib-0128] Herbec, A. , Brown, J. , Tombor, I. , Michie, S. , & West, R. (2014). Pilot randomized controlled trial of an internet‐based smoking cessation intervention for pregnant smokers (‘MumsQuit’). Drug and Alcohol Dependence, 140, 130–136. 10.1016/j.drugalcdep.2014.04.010 24811202 PMC4067748

[nop21822-bib-0129] Herring, S. J. , Albert, J. J. , Darden, N. , Bailer, B. , Cruice, J. , Hassan, S. , Bennett, G. G. , Goetzl, L. , Yu, D. , Kilby, L. M. , & Foster, G. D. (2019). Targeting pregnancy‐related weight gain to reduce disparities in obesity: Baseline results from the Healthy Babies trial. Contemporary Clinical Trials, 87, 105822. 10.1016/j.cct.2019.105822 31400513 PMC7265899

[nop21822-bib-0130] Himes, K. P. , Donovan, H. , Wang, S. , Weaver, C. , Grove, J. R. , & Facco, F. L. (2017). Healthy beyond pregnancy, a web‐based intervention to improve adherence to postpartum care: Randomized controlled feasibility trial. JMIR Human Factors, 4(4), e26. 10.2196/humanfactors.7964 29017990 PMC5654734

[nop21822-bib-0131] Hirshberg, A. , Downes, K. , & Srinivas, S. (2018). Comparing standard office‐based follow‐up with text‐based remote monitoring in the management of postpartum hypertension: A randomised clinical trial. BMJ Quality & Safety, 27(11), 871–877. 10.1136/bmjqs-2018-007837 29703800

[nop21822-bib-0012] Higgins, T. , Larson, E. , & Schnall, R. (2017). Unraveling the meaning of patient engagement: A concept analysis. Patient Education and Counseling, 100(1), 30–36. 10.1016/j.pec.2016.09.002 27665500

[nop21822-bib-0132] Holm, K. G. , Brodsgaard, A. , Zachariassen, G. , Smith, A. C. , & Clemensen, J. (2019). Parent perspectives of neonatal tele‐homecare: A qualitative study. Journal of Telemedicine and Telecare, 25(4), 221–229. 10.1177/1357633X18765059 29792079

[nop21822-bib-0133] Isetta, V. , Lopez‐Agustina, C. , Lopez‐Bernal, E. , Amat, M. , Vila, M. , Valls, C. , Navajas, D. , & Farre, R. (2013). Cost‐effectiveness of a new internet‐based monitoring tool for neonatal post‐discharge home care. Journal of Medical Internet Research, 15(2), e38. 10.2196/jmir.2361 23419609 PMC3636285

[nop21822-bib-0134] Jefferson, U. T. , Zachary, I. , & Majee, W. (2019). Employing a user‐centered design to engage mothers in the development of a mhealth breastfeeding application. CIN:Computers Informatics Nursing, 37(10), 522–531. 10.1097/CIN.0000000000000549 31414995

[nop21822-bib-0135] Ke, J. X. C. , George, R. B. , Wozney, L. , & Munro, A. (2021). Perioperative mobile application for mothers undergoing Cesarean delivery: A prospective cohort study on patient engagement. Canadian Journal of Anesthesia/Journal Canadien d’anesthésie, 68(4), 505–513. 10.1007/s12630-020-01907-x PMC7794079 33420678

[nop21822-bib-0013] Kelders, S. M. , Kip, H. , & Greeff, J. (2020). Psychometric evaluation of the TWente engagement with Ehealth technologies scale (TWEETS): Evaluation study. Journal of Medical Internet Research, 22(10), e17757. 10.2196/17757 33021487 PMC7576538

[nop21822-bib-0014] Kelders, S. M. , van Zyl, L. E. , & Ludden, G. D. S. (2020). The concept and components of engagement in different domains applied to eHealth: A systematic scoping review. Frontiers in Psychology, 11, 926. 10.3389/fpsyg.2020.00926 32536888 PMC7266981

[nop21822-bib-0136] Kennelly, M. A. , Ainscough, K. , Lindsay, K. , Gibney, E. , Mc Carthy, M. , & McAuliffe, F. M. (2016). Pregnancy, exercise and nutrition research study with smart phone app support (Pears): Study protocol of a randomized controlled trial. Contemporary Clinical Trials, 46, 92–99. 10.1016/j.cct.2015.11.018 26625980

[nop21822-bib-0015] Kiwanuka, F. , Nanyonga, R. C. , Sak‐Dankosky, N. , Muwanguzi, P. A. , & Kvist, T. (2021). Nursing leadership styles and their impact on intensive care unit quality measures: An integrative review. Journal of Nursing Management, 29(2), 133–142. 10.1111/jonm.13151 32881169

[nop21822-bib-0016] Korstjens, I. (2021). The paradoxes of communication and collaboration in maternity care: A video‐reflexivity study with professionals and parents. Women and Birth, 9, 145–153. 10.1016/j.wombi.2020.01.014 32063528

[nop21822-bib-0137] Krishnamurti, T. , Davis, A. L. , Wong‐Parodi, G. , Fischhoff, B. , Sadovsky, Y. , & Simhan, H. N. (2017). Development and testing of the MyHealthyPregnancy app: A behavioral decision research‐based tool for assessing and communicating Pregnancy risk. JMIR MHealth and UHealth, 5(4), e42. 10.2196/mhealth.7036 28396302 PMC5404142

[nop21822-bib-0017] Kyngäs, H. , Mikkonen, K. , & Kääriäinen, M. (2020a). Deductive content analysis. In The application of content analysis in nursing science research (pp. 23–30). Springer.

[nop21822-bib-0018] Kyngäs, H. , Mikkonen, K. , & Kääriäinen, M. (2020b). Inductive content analysis. In The application of content analysis in nursing science research (pp. 13–21). Springer.

[nop21822-bib-0019] Labrie, N. H. M. , van Veenendaal, N. R. , Ludolph, R. A. , Ket, J. C. F. , van der Schoor, S. R. D. , & van Kempen, A. A. M. W. (2021). Effects of parent‐provider communication during infant hospitalization in the NICU on parents: A systematic review with meta‐synthesis and narrative synthesis. Patient Education and Counseling, 104(7), 1526–1552. 10.1016/j.pec.2021.04.023 33994019

[nop21822-bib-0138] Ledford, C. J. W. , Womack, J. J. , Rider, H. A. , Seehusen, A. B. , Conner, S. J. , Lauters, R. A. , & Hodge, J. A. (2017). Unexpected effects of a system‐distributed mobile application in maternity care: A randomized controlled trial. Health Education and Behavior, 45(3), 323–330. 10.1177/1090198117732110 28918669

[nop21822-bib-0020] Lieu, T. A. , Gizzi, E. , & Lee, E. R. (2021). Reinventing pediatrics through video care first. JAMA Pediatrics, 175(3), 232. 10.1001/jamapediatrics.2020.4684 33136140

[nop21822-bib-0021] Liu, S. (2016). Delivering on digital: A review of virtual health literature in perinatal care . (Master's thesis). Simon Fraser University, British Columbia 82.

[nop21822-bib-0139] Marko, K. I. , Krapf, J. M. , Meltzer, A. C. , Oh, J. , Ganju, N. , Martinez, A. G. , Sheth, S. G. , & Gaba, N. D. (2016). Testing the feasibility of remote patient monitoring in prenatal care using a mobile app and connected devices: A prospective observational trial. JMIR Research Protocols, 5(4), e200. 10.2196/resprot.6167 27864167 PMC5135734

[nop21822-bib-0022] Millenson, M. L. , & Macri, J. (2012). Will the affordable care act move patient‐centeredness to center stage? (552112012–001) [data set]. American Psychological Association. 10.1037/e552112012-001

[nop21822-bib-0023] Mol, A. (2018). The logic of care: Health and the problem of patient choice (p. 19). Routledge: Taylor & Francis Group.

[nop21822-bib-0024] Molenaar, J. , Korstjens, I. , Hendrix, M. , de Vries, R. , & Nieuwenhuijze, M. (2018). Needs of parents and professionals to improve shared decision‐making in interprofessional maternity care practice: A qualitative study. Birth, 45(3), 245–254. Scopus 10.1111/birt.12379. 30051527 10.1111/birt.12379

[nop21822-bib-0025] Molenaar, J. M. , Lips, S. R. , Teunissen, P. W. , Vermeulen, G. , & Schuitmaker‐Warnaar, T. J. (2020). Creating togetherness in a historically divided maternity care system / Zusammengehörigkeit in einem historisch gespaltenen geburtshilflichen Versorgungssystem herstellen. International Journal of Health Professions, 7(1), 33–44. 10.2478/ijhp-2020-0004

[nop21822-bib-0140] Naughton, F. , Jamison, J. , & Sutton, S. (2013). Attitudes towards SMS text message smoking cessation support: A qualitative study of pregnant smokers. Health Education Research, 28(5), 911–922. 10.1093/her/cyt057 23640985

[nop21822-bib-0026] Nelson, G. A. , & Holschuh, C. (2021). Evaluation of telehealth use in prenatal Care for Patient and Provider Satisfaction: A step toward reducing barriers to care. The Journal for Nurse Practitioners, 17(4), 481–484. 10.1016/j.nurpra.2020.12.026

[nop21822-bib-0141] O’Brien, E. , Rauf, Z. , Alfirevic, Z. , & Lavender, T. (2013). Women's experiences of outpatient induction of labour with remote continuous monitoring. Midwifery, 29(4), 325–331. 10.1016/j.midw.2012.01.014 23159160

[nop21822-bib-0027] Oberschmidt, K. , Grünloh, C. , Nijboer, F. , & van Velsen, L. (2022). Best practices and lessons learned for action research in eHealth design and implementation: Literature review. Journal of Medical Internet Research, 24(1), e31795. 10.2196/31795 35089158 PMC8838546

[nop21822-bib-0028] Ouzzani, M. , Hammady, H. , Fedorowicz, Z. , & Elmagarmid, A. (2016). Rayyan—A web and mobile app for systematic reviews. Systematic Reviews, 5(1), 210. 10.1186/s13643-016-0384-4 27919275 PMC5139140

[nop21822-bib-0029] Page, M. J. , McKenzie, J. E. , Bossuyt, P. M. , Boutron, I. , Hoffmann, T. C. , Mulrow, C. D. , Shamseer, L. , Tetzlaff, J. M. , Akl, E. A. , Brennan, S. E. , Chou, R. , Glanville, J. , Grimshaw, J. M. , Hróbjartsson, A. , Lalu, M. M. , Li, T. , Loder, E. W. , Mayo‐Wilson, E. , McDonald, S. , … Moher, D. (2021). The PRISMA 2020 statement: An updated guideline for reporting systematic reviews. International Journal of Surgery, 88(105), 906. 10.1016/j.ijsu.2021.105906 33789826

[nop21822-bib-0142] Payakachat, N. , Rhoads, S. , McCoy, H. , Dajani, N. , Eswaran, H. , & Lowery, C. (2020). Using mHealth in postpartum women with pre‐eclampsia: Lessons learned from a qualitative study. International Journal of Gynecology & Obstetrics, 149(3), 339–346. 10.1002/ijgo.13134 32119129 PMC7239748

[nop21822-bib-0030] Pham, M. T. , Rajić, A. , Greig, J. D. , Sargeant, J. M. , Papadopoulos, A. , & McEwen, S. A. (2014). A scoping review of scoping reviews: Advancing the approach and enhancing the consistency. Research Synthesis Methods, 5(4), 371–385. 10.1002/jrsm.1123 26052958 PMC4491356

[nop21822-bib-0031] Phillippi, J. C. , Holley, S. L. , Payne, K. , Schorn, M. N. , & Karp, S. M. (2016). Facilitators of prenatal care in an exemplar urban clinic. Women and Birth, 29(2), 160–167. 10.1016/j.wombi.2015.09.007 26530714

[nop21822-bib-0143] Platonos, K. , Aloysius, A. , Banerjee, J. , & Deierl, A. (2018). Integrated family delivered care project: Parent education programme. Journal of Neonatal Nursing, 24(1), 29–34. 10.1016/j.jnn.2017.11.008

[nop21822-bib-0144] Rhoads, S. J. , Serrano, C. I. , Lynch, C. E. , Ounpraseuth, S. T. , Heath Gauss, C. , Payakachat, N. , Lowery, C. L. , & Eswaran, H. (2017). Exploring implementation of m‐health monitoring in postpartum women with hypertension. Telemedicine and E‐Health, 23(10), 833–841. 10.1089/tmj.2016.0272 28475431 PMC5651969

[nop21822-bib-0032] Richardson, B. , Goldberg, L. , Aston, M. , & Campbell‐Yeo, M. (2018). eHealth versus. Equity: Using a feminist poststructural framework to explore the influence of perinatal eHealth resources on health equity. Journal of Clinical Nursing, 27(21–22), 4224–4233. 10.1111/jocn.14592 29964310

[nop21822-bib-0145] Ridgeway, J. L. , LeBlanc, A. , Branda, M. , Harms, R. W. , Morris, M. A. , Nesbitt, K. , Gostout, B. S. , Barkey, L. M. , Sobolewski, S. M. , Brodrick, E. , Inselman, J. , Baron, A. , Sivly, A. , Baker, M. , Finnie, D. , Chaudhry, R. , & Famuyide, A. O. (2015). Implementation of a new prenatal care model to reduce office visits and increase connectivity and continuity of care: Protocol for a mixed‐methods study. BMC Pregnancy and Childbirth, 15(1), 323. 10.1186/s12884-015-0762-2 26631000 PMC4668747

[nop21822-bib-0033] Scimago Lab . (2021). Scimago journal & country rank. Scopus® Retrieved June 10, 2022, from: https://www.scimagojr.com/journalrank.php?area=2900

[nop21822-bib-0146] Shorey, S. , Yang, Y. Y. , & Dennis, C. L. (2018). A mobile health app‐based postnatal educational program (home‐but not alone): Descriptive qualitative study. Journal of Medical Internet Research, 20(4), 1. 10.2196/jmir.9188 PMC5934535 29674314

[nop21822-bib-0034] Sfantou, D. , Laliotis, A. , Patelarou, A. , Sifaki‐ Pistolla, D. , Matalliotakis, M. , & Patelarou, E. (2017). Importance of leadership style towards quality of care measures in healthcare settings: A systematic review. Healthcare, 5(4), 73. 10.3390/healthcare5040073 29036901 PMC5746707

[nop21822-bib-0147] Soltani, H. , Duxbury, A. M. S. , Arden, M. A. , Dearden, A. , Furness, P. J. , & Garland, C. (2015). Maternal obesity management using mobile technology: A feasibility study to evaluate a text messaging based complex intervention during pregnancy. Journal of Obesity, 2015, 814830. 10.1155/2015/814830 25960889 PMC4415456

[nop21822-bib-0148] Spargo, P. , & de Vries, N. K. (2018). ‘Babble’: A smartphone app for parents who have a baby in the neonatal unit: Babble. Journal of Paediatrics and Child Health, 54(2), 121–123. 10.1111/jpc.13817 29417669

[nop21822-bib-0035] Stelwagen, M. A. , van Kempen, A. A. M. W. , Westmaas, A. , Blees, Y. J. , & Scheele, F. (2020). Integration of maternity and neonatal care to empower parents. Journal of Obstetric, Gynecologic & Neonatal Nursing, 49(1), 65–77. 10.1016/j.jogn.2019.11.003 31809695

[nop21822-bib-0149] Strand, A. S. , Johnsson, B. , Hena, M. , Magnusson, B. , & Hallström, I. K. (2021). Developing eHealth in neonatal care to enhance parents’ self‐management. Scandinavian Journal of Caring Sciences, 36(4), 969–977 https://pubmed.ncbi.nlm.nih.gov/33950534/ 33950534 10.1111/scs.12994

[nop21822-bib-0036] Tavory, I. , & Timmermans, S. (2014). Abductive analysis: Theorizing qualitative. Research. The University of Chicago Press.

[nop21822-bib-0037] Tricco, A. C. , Lillie, E. , Zarin, W. , O'Brien, K. K. , Colquhoun, H. , Levac, D. , Moher, D. , Peters, M. D. J. , Horsley, T. , Weeks, L. , Hempel, S. , Akl, E. A. , Chang, C. , McGowan, J. , Stewart, L. , Hartling, L. , Aldcroft, A. , Wilson, M. G. , Garritty, C. , … Straus, S. E. (2018). PRISMA extension for scoping reviews (PRISMA‐ScR): Checklist and explanation. Annals of Internal Medicine, 169(7), 467. 10.7326/M18-0850 30178033

[nop21822-bib-0150] Triebwasser, J. E. , Janssen, M. K. , Hirshberg, A. , & Srinivas, S. K. (2020). Successful implementation of text‐based blood pressure monitoring for postpartum hypertension. Pregnancy Hypertension, 22, 156–159. 10.1016/j.preghy.2020.09.001 32980623

[nop21822-bib-0038] van den Heuvel, J. F. , Groenhof, T. K. , Veerbeek, J. H. , van Solinge, W. W. , Lely, A. T. , Franx, A. , & Bekker, M. N. (2018). eHealth as the next‐generation perinatal care: An overview of the literature. Journal of Medical Internet Research, 20(6), e202. 10.2196/jmir.9262 29871855 PMC6008510

[nop21822-bib-0152] van der Wulp, N. Y. , Hoving, C. , Eijmael, K. , Candel, M. J. , van Dalen, W. , & De Vries, H. (2014). Reducing alcohol use during pregnancy via health counseling by midwives and internet‐based computer‐tailored feedback: A cluster randomized trial. Journal of Medical Internet Research, 16(12), e274. 10.2196/jmir.3493 25486675 PMC4275508

[nop21822-bib-0039] van Veenendaal, N. R. , Auxier, J. N. , van der Schoor, S. R. D. , Franck, L. S. , Stelwagen, M. A. , de Groof, F. , van Goudoever, J. B. , Eekhout, I. E. , de Vet, H. C. W. , Axelin, A. , & van Kempen, A. A. M. W. (2021). Development and psychometric evaluation of the CO‐PARTNER tool for collaboration and parent participation in neonatal care. PLoS One, 16(6), e0252074. 10.1371/journal.pone.0252074 34106929 PMC8189480

[nop21822-bib-0151] Valencia, S. , Callinan, L. , Shic, F. , & Smith, M. (2020). Evaluation of the MoMba live long remote smoking detection system during and after pregnancy: Development and usability study. JMIR MHealth and UHealth, 8(11), e18809. 10.2196/18809 33231550 PMC7723738

[nop21822-bib-0153] Wierckx, A. , Shahid, S. , & Al Mahmud, A. (2014). Babywijzer: An application to support women during their pregnancy. In M. Jones (Ed.), CHI ’14 Extended abstracts on human factors in computing systems (pp. 1333–1338). Association for Computing Machinery. 10.1145/2559206.2581179

[nop21822-bib-0154] Willcox, J. C. , Campbell, K. J. , McCarthy, E. A. , Wilkinson, S. A. , Lappas, M. , Ball, K. , Fjeldsoe, B. , Griffiths, A. , Whittaker, R. , Maddison, R. , Shub, A. , Pidd, D. , Fraser, E. , Moshonas, N. , & Crawford, D. A. (2015). Testing the feasibility of a mobile technology intervention promoting healthy gestational weight gain in pregnant women (txt4two)—Study protocol for a randomised controlled trial. Trials, 16(1), 209. 10.1186/s13063-015-0730-1 25947578 PMC4426547

[nop21822-bib-0040] World Health Organization . (2016). Monitoring and evaluating digital health interventions: A practical guide to conducting research and assessment. World Health Organization https://apps.who.int/iris/handle/10665/252183

[nop21822-bib-0041] World Health Organization . (2018). Classification of digital health interventions v1.0: A shared language to describe the uses of digital technology for health. (WHO/RHR/18.06). World Health Organization Retrieved from: https://apps.who.int/iris/bitstream/handle/10665/260480/WHO‐RHR‐18.06‐eng.pdf

[nop21822-bib-0042] Wreesmann, W. W. , Lorié, E. S. , van Veenendaal, N. R. , van Kempen, A. A. M. W. , Ket, J. C. F. , & Labrie, N. H. M. (2021). The functions of adequate communication in the neonatal care unit: A systematic review and meta‐synthesis of qualitative research. Patient Education and Counseling, 104(7), 1505–1517. 10.1016/j.pec.2020.11.029 33341329

[nop21822-bib-0155] Yee, L. M. , Leziak, K. , Jackson, J. , Strohbach, A. , Saber, R. , Niznik, C. M. , & Simon, M. A. (2021). Patient and provider perspectives on a novel mobile health intervention for low‐income pregnant women with gestational or type 2 Diabetes mellitus. Journal of Diabetes Science and Technology, 15(5), 1121–1133. 10.1177/1932296820937347 32627582 PMC8442184

