# Supplementary material for: Patient engagement practice within perinatal eHealth: A scoping review
Source: Nurs Open. 2023 May 21;10(8):4971–84. doi: 10.1002/nop2.1822 (PMC10333891; doi:10.1002/nop2.1822)
Supplement: Supplementary file 1 — Data S1. [file NOP2-10-4971-s001.docx]

Table of Contents

[S1: Characteristics of Primary Sources 2](#_Toc105684134)

[S2: Reference List of Included Sources 43](#_Toc105684135)

[S3: Behavior Change Theory Use 54](#_Toc105684136)

[S4: Table Showing Missing Commitment and Therapeutic Alliance Attributes 56](#_Toc105684137)

# S1: Characteristics of Primary Sources

**Table S1**

Characteristics of Primary Sources

| Authors, year, country | Study Design | Aim | Target user/Setting | Program structure/devices | WHO DHI Categories | Engagement Outcome | Modalities of eHealth |
| --- | --- | --- | --- | --- | --- | --- | --- |
| Abbass-Dick et al. (2017)  *Canada* | Three-phase exploratory study | Designing and pilot testing an interactive eHealth breastfeeding co-parenting resource developed to target both mothers and fathers | LOW-RISK post-partum  Pregnant women/ new mothers and their partners  (*N* = 149) | Website for desktop and mobile use with the following components:  Education materials (documents, videos, and games) Quizzes Father specific information Mother specific information Health resource links | - Targeted client communication - On-demand information services | N/A | - Goal setting - On-demand information and resources - On-demand tailored information - Online games |
| Abroms et al. (2017)  *USA* | Randomized control trial | Investigating the supplemental effect of adding a smoking-cessation text messaging program, Quit4baby to an established text messaging program, Text4baby | HIGH-RISK  antenatal  Pregnant women who are smokers <32 GW  (*N* = 497) | SMS program for mobile use | - Targeted client communication - Client to client communication - On-demand information services | N/A | - Goal setting - On-demand information and resources - On-demand tailored information - Peer support - Tailored feedback |
| Banerjee et al.  (2020)  *UK* | Program report | Integrated Family Delivered Care (IFDC) programme was to improve infant health outcomes and parent experience through education and competency- based training | HIGH-RISK neonatal  Families  (*N*= 89) | Family Integrated Care (FICare) App:  Digital journal for parental tasks and close time spent with infants;  Data collected in journal can be used in FICare peer and professional coaching | - Personal health tracking - On-demand information services | Percentage of parents using App | - Digital log keeping - On-demand information and resources |
| Baron et al. (2018) *USA* | Qualitative evaluation | Exploring the perspectives of patients, RNs, and other providers regarding a new prenatal connected care model | LOW-RISK antenatal  Pregnant women  <13 GW  18-36 yoa  (*N* = 41)   RNs  (*N* =10)  Other HCPs (*N* = 17) | 6 virtual visits (phone/online  communication with nurses);  Fetal Doppler and sphygmomanometer home  monitoring devices;  Online community of pregnant women; Medical records for submitting parameter data to nurses if they wish to use (can also send  data by phone) | - Targeted client communication - Client to client communication - Personal health tracking - On-demand information services | N/A | - Decision support - On-demand information and resources - On-demand tailored information - Peer support - Remote communication - Self-monitoring - Tailored feedback |
| Baruth et al. (2019) *USA* | Feasibility randomized retrospective control trial | Assessing the feasibility of a pilot behavioral lifestyle intervention | LOW-RISK antenatal  Pregnant women  14-20 GW  (*N* = 82) | Actigraphy (GT3x Actigraphy); Fitbit, & scale; study website;  private, moderated Facebook page | - Targeted client communication - Client to client communication - Personal health tracking - On-demand information services | Process evaluation measures regarding usage of study components | - Goal setting - On-demand information and resources - Peer support - Remote communication - Self-monitoring - Tailored feedback |
| Bower et al. (2005)  *UK* | Program report | N/A | HIGH-RISK neonatal  Staff use the system; Users included parents of babies on the unit and clinical audit personnel | Nursing and medical staff involved in the care of patients in the unit enter information about the patient including information on procedures that their baby may be undergoing;  Parent users access the system at the unit or from home by the Internet using a personal password; Parents sign off on the system for approval of newborn care based on information held on the system | - On-demand information services | N/A | - Decision support - On-demand tailored information |
| Demirci et al.  (2020)  *USA* | Feasibility study phase of a Randomized controlled trial | Examining user engagement with and feedback on a theory-grounded SMS text messaging intervention intended to prevent perceived insufficient milk | LOW-RISK  antenatal & postpartum  Pregnant women  (*N*=247) | Mobile, semiautomated text message-based Intervention too prevent perceived Low or insufficient milK supply (MILK) SMS text message system | - Targeted client communication - On-demand information services | Volume of using the system by sending query or keyword texting | - On-demand tailored information - Remote communication - Tailored feedback |
| Carrilho et al. (2019)  *Brazil* | Observational, exploratory, descriptive study | Evaluating pregnant women’s perception about the communicability of birth-plan preparation using a mobile App | LOW-RISK antenatal  Pregnant women  18-39 yoa  (*N* = 11) | My Prenatal Care App:  Sequential modules for planning throughout pregnancy & birth; digital record of women’s desires and expectations;  App information is accessible to healthcare providers remotely; Tailored feedback | - Targeted client communication - Personal health tracking | N/A | - Decision support - Self-monitoring - Tailored feedback |
| Choi et al. (2015)  *USA* | Pilot Randomized controlled trial | Testing a 12-week mobile health (mHealth) physical activity intervention for feasibility and potential efficacy | LOW-RISK antenatal  Pregnant women  10-20 GW 18-40 yoa  (*N* = 30) | Mobile phone App:  Fitbit monitoring | - Targeted client communication - Personal health tracking - On-demand information services | Daily messages and using the daily diary through the mobile App | - Goal setting - On-demand information and resources - On-demand tailored information - Self-monitoring |
| Cramer et al. (2018)  *USA* | Two group experimental design (Pilot) | Assessing the feasibility of a tailored smartphone platform intervention combined with CHW reinforcement among rural pregnant women | HIGH-RISK antenatal  Pregnant women  > 24 GW  all ages (minors needed parental consent)  (*N* =98) | Smartphone App:  SMS messaging, tailored feedback, and on-demand information and resources | - Targeted client communication - On-demand information services | N/A | - On-demand information and resources - Remote communication - Tailored feedback |
| Dalton et al. (2018)  *Australia* | Retrospective review of quantitative and qualitative evaluation of Health-e Babies App | Analyzing the characteristics of the participants who did not complete the study requirements in an attempt to identify potential barriers associated with the implementation of a pregnancy App | LOW-RISK antenatal  Pregnant women =>18 years 10-14 GW  (*N* = 85) | Android App:  Tailored feedback,  on-demand information and resources,  peer-support, & decision support | - Targeted client communication - Client to client communication - On-demand information services | Engagement and use of the Health-e Babies App was measured by the completion of a questionnaire and downloaded data from participant’s phones | - Decision support - On-demand information and resources - Peer support - Tailored feedback |
| Danbjørg et al. (2014) *Denmark* | Participatory design pilot study | Exploring to what extent the nurses consider that the IT solution fits into their working routines, families’ experience of the IT solution as a response to their follow-up support needs, and the experience of nurses and families in relation to communicating online | LOW-RISK neonatal  Families being discharged >= 24 hours (*N* =10)    Nurses  (*N* = 15) | App & website; Communication platform: text messages, pictures, and videos can be exchanged; Information database including documents & multimedia for education;  Automated tailored SMS messages | - Targeted client communication - Personal health tracking - On-demand information services | N/A | - Decision support - On-demand information and resources - On-demand tailored information - Remote communication - Self-monitoring - Tailored feedback |
| Danbjørg et al. (2015)  *Denmark* | Participatory design | Exploring how postnatal parents experienced the use of telemedicine following early discharge from hospital (i.e. 24 hours after childbirth) by investigating if they consider that their postnatal needs are met, and whether or not they experience a sense of security and parental self-efficacy | LOW-RISK neonatal  Families experience-ing early discharge  Parent sets  (*N* = 10)    Mothers only  (*N* = 17)  Fathers only  (*N* = 1) | App & website; Communicat-ion platform: text messages, pictures, and videos can be exchanged; Information database including documents & multimedia for education;  Automated tailored SMS messages | - Targeted client communication - Personal health tracking - On-demand information services | N/A | - Decision support - On-demand information and resources - On-demand tailored information - Remote communication - Self-monitoring - Tailored feedback |
| Davis et al. (2018)  *Australia* | Randomized control design with qualitative components (Protocol) | Examining the effectiveness of the Eating4Two smartphone App in assisting pregnant women to limit GWG; Comparing childbirth outcomes and satisfaction with antenatal care and examining the way that relative advantage and disadvantage might influence engagement with the acceptability of the intervention | LOW-RISK & HIGH-RISK antenatal  Pregnant women  < 15 GW  Goal (*N* = 1330) | Eating4Two smartphone App:  library of information, tailored feedback, decision-support functionality, and self-monitoring through digital journal | - Targeted client communication - Personal health tracking - On-demand information services | N/A | - Decision support - On-demand information and resources - Self-monitoring - Tailored feedback |
| de Mooij et al. (2018)  USA | Qualitative Design and protype development | Designing and developing a PCC model that includes novel care tools for women | LOW-RISK antenatal  Pregnant women  <13 GW  18-36 yoa  (N = 300) | 6 virtual visits (phone/online  communication with nurses);  Fetal Doppler and sphygmomanometer home  monitoring devices; Online community of pregnant women; Medical records for submitting parameter data to nurses if they wish to use (can also send  data by phone) | - Targeted client communication - Client to client communication - Personal health tracking - On-demand information services | N/A | - Decision support - On-demand information and resources - On-demand tailored information - Peer support - Remote communication - Self-monitoring - Tailored feedback |
| Demment et al. (2014)  *USA* | Randomized controlled trial | (1) Characterizing how pregnant women engaged with features of an online intervention to prevent excessive GWG;  (2) Identifying demographic and weight status subgroups of women within the sample, and (3) Examining differences in use of intervention features across the demographic and weight status subgroups | HIGH-RISK antenatal  Pregnant women  => 20 GW  (*N* = 1689) | Weight-gain tracker; Health-related articles & blogs;  Physical activity & diet goal-setting tools;  Links to local resources | - Targeted client communication - Client to client communication - Personal health tracking - On-demand information services | Level of engagement in eHealth features:  Amount  Consistency  Patterns | - Decision support - Goal setting - On-demand information and resources - Peer support - Remote communication - Self-monitoring - Tailored feedback |
| Doherty et al. (2018)  *UK* | Focus group design and qualitative report | Identifying requirements for a mobile App for deployment within a public health service, explores concept development with users, and performance of an iterative prototype evaluation | HIGH-RISK antenatal  Pregnant women  (*N* = 46)  GPs  (*N* = 2)  Clinical psychologist  (*N =* 1)  Child and Adolescent psychiatrist  (*N =* 1)  Maternal and child health researchers (*N* = 2) | BrightSelf, a mobile App & clinical  interface for the self-report of psychological  well-being in pregnancy | - Personal health tracking - On-demand information services | N/A | - Decision support - On-demand information and resources - Self-monitoring |
| Doherty et al. (2019)  *UK* | Feasibility study | A Longitudinal de- ployment of a mobile application for the self-report of psychological wellbeing in antenatal clinical practice; an interdisciplinary undertaking involving HCI and public health researchers, pregnant women and a variety of health professionals including midwives | HIGH-RISK antenatal  Pregnant women  GW 12/14  (*N* = 254) | BrightSelf, a mobile App & and Ecological Momentary Assessment (EMA) logging for the self-report of psychological  well-being in pregnancy | - Targeted client communication - Personal health tracking - On-demand information services | - Engagement as Self-Report - Engagement as Protocol Adherence - Engagement by Time Spent - Engagement by Population & Wellbeing - Engagement & Wellbeing/Users in Distress - Subjective Engagement (Qualitative) | - EMA Logging - On-demand information - and resources - Remote communication - Self-monitoring - Tailored feedback |
| Doherty et al.  (2020)  *UK* | Feasibility study | A Longitudinal de- ployment of a mobile application for the self-report of psychological wellbeing in antenatal clinical practice; an interdisciplinary undertaking involving HCI and public health researchers, pregnant women and a variety of health professionals including midwives | HIGH-RISK antenatal  Pregnant women  GW 12/14  (*N* = 254) | BrightSelf, a mobile App & and Ecological Momentary Assessment (EMA) logging for the self-report of psychological  well-being in pregnancy | - Targeted client communication - Personal health tracking - On-demand information services | Process evaluation of four patient engagement attributes | - EMA Logging - On-demand information   and resources   - Remote communication - Self-monitoring - Tailored feedback |
| Dougall et al. (2020)  *USA* | Report on two multicentre, non- masked, parallel group, randomised controlled trials  (Protocol) | Investigating self- monitoring of BP during pregnancy in the UK in secondary care maternity units | HIGH-RISK  Maternity  Pregnant women  Pilot test (*N*= 50)  RCT (*N*=2262) | Microlife WatchBP Home  Texting and Application based telemonitoring system | - Targeted client communication - Personal health tracking | Fidelity to monitoring schedule | - Self-monitoring - Tailored Feedback |
| Fontein-Kuipers et al.  (2016)  *The Netherlands* | Non-randomized pre-post intervention study with a sequential control and experimental group | Evaluating the effect of the intervention  WazzUp Mama?! on antenatal maternal distress by examining the changes across time in maternal distress among healthy pregnant women who received the antenatal intervention WazzUp Mama?! and healthy pregnant women who received antenatal care-as-usual, and the differences between these groups. | HIGH-RISK  antenatal  Pregnant women  (*N* = 433) | A web-based program that enables digital logging of mental state and other health and demographic information. The program gives tailored feedback about mental wellness and is used for screening for mental health state and best care plan fit. | - Targeted client communication - Personal health tracking | N/A | - Decision Support - Digital Patient Logging - Tailored Feedback |
| Franck et al.  (2019)  *USA* | Exploratory multi-site quasi-experimental study | Determining the feasibility and acceptability of mobile application technology to gather data about parent involvement in the care of NICU infants in the usual FCC and in the mFICare parent intervention | HIGH-RISH neonatal | We3health^TM^ mobile application that support education, communication, parental support, and data collection of parent involvement in the care of their infants in the NICU | - Client to client communication - Personal health tracking - On-demand information services | Parent involvement in care of infant in NICU | - Decision support - Digital log keeping - On-demand information and resources - On-demand tailored information - Peer Support - Remote Communication |
| Frize et al. (2011)  *Canada* | Pro-type evaluation | Exploring the design options for the development of a combined physician-parent decision support tool for a neonatal intensive care unit (NICU), Physician and Parent Decision Support (PPADS) | HIGH-RISK neonatal  Physician and Nurse decision support specialists, neonatal care experts | Platform (PPADS) includes clinical updates and predictions of clinical outcomes;  Two-way communication between parents and physicians | - Targeted client communication - On-demand information services | N/A | - Decision support - On-demand tailored information - Remote communication - Tailored feedback |
| Frize et al. (2013)  *Canada* | Pro-type usability (next level) evaluation | Testing usability of Physician and Parent Decision Support (PPADS) system | HIGH-RISK neonatal  Parents with history of infant in NICU  (*N* = 8)  Neonatol-ogists  (*N* = 5) | Platform (PPADS) includes clinical updates and predictions of clinical outcomes;  Two-way communicat-ion between parents and physicians | - Targeted client communication - On-demand information services | N/A | - Decision support - On-demand tailored information - Remote communication - Tailored feedback |
| Garfield et al.  (2016)  *USA* | Randomized controlled trial | Determining whether parents of Very Low Birth Weight (VLBW) infants in the Neonatal Intensive Care Unit (NICU) transitioning home with the NICU-2-Home smartphone application have greater parenting self- efficacy, are better prepared for discharge and have shorter length of stay (LOS) than control parents | HIGH-RISK neonatal  Parents  (*N* = 91) | Android smartphone & NICU-2-Home App: Digital patient log for note keeping; On demand tailored and generic information and resources; self-monitoring | - Personal health tracking - On-demand information services | N/A | - Digital log keeping - On-demand information and resources - On-demand tailored information - Self-monitoring |
| Garne et al. (2016)  *Denmark* | Participatory design; qualitative study | Identifying parental needs when wanting to provide neonatal home care supported by telemedicine | LOW-RISK neonatal  Parents  (*N* = 19) | Tele-Health application;  Weigh scale;  Digital parent assessment log | - Targeted client communication - Personal health tracking - On-demand information services | N/A | - Decision support - Digital log keeping - On-demand information and resources - On-demand tailored information - Remote communication - Self-monitoring - Tailored feedback |
| Garne et al. (2017)  *Denmark* | Participatory design; qualitative study | Developing a clinical telehealth service for neonatal homecare | LOW-RISK neonatal  Healthcare staff and parents  (*N* = not specified) | Tele-Health App;  Weigh scale;  Digital parent assessment log | - Targeted client communication - Personal health tracking - On-demand information services | N/A | - Decision support - On-demand information and resources - On-demand tailored information - Remote communication - Self-monitoring - Tailored feedback |
| Gibson et al.  (2021)  *New Zealand* | Qualitative Study | (1) Identifying and exploring (from the parents’ own perspective) experiences in a neonatal setting, specifically in a Level II+ unit, and (b) Assessing the feasibility and usefulness of the Babble app across the neonatal experience | HIGH-RISK  neonatal  Mothers of newborns admitted to the NICU  (*N* = 8) | The Babble application includes resources, information and journal entries about parents time in the NICU and for the time after going home | - Personal health tracking - On-demand information services | Information access  (how did you learn about your baby and the care they were provided?) | - Digital log keeping - On-demand information and resources |
| Globus et al. (2016)  *Israel* | Pre & post intervention evaluation study | Evaluating the use of SMS technology and to assess its impact on the parents and the nursing staff | LOW-RISK neonatal  Parents  (*N* = 178) | Updates on clinical status and events sent to parents by SMS; Decision support; & Tailored feedback | - Targeted client communication | N/A | - Decision support - Tailored feedback |
| Graham et al. (2014)  *USA* | Formative research design | Investigating the development of an e-intervention to prevent excessive GWG | HIGH-RISK antenatal  Pregnant women  18-35 yoa  (*N* = 160) | Weight-gain tracker; Health-related articles & blogs;  Physical activity & diet goal-setting tools;  Links to local resources | - Targeted client communication - Client to client communication - Personal health tracking - On-demand information services | Use of each eHealth feature | - Decision support - Goal setting - On-demand information and resources - Peer support - Remote communication - Self-monitoring - Tailored feedback |
| Gu et al.  (2014)  *China* | Conference report | Reporting on the proposed eHealth technology, role-based knowledge management system | HIGH-RISK antenatal  Pregnant women  (*N* ~ 100) | Patient Management mobile use system for high-risk pregnancy care;  Mobile-based knowledge management; Maternal health data management; | - Targeted client communication - Personal health tracking | N/A | - Decision support - Self-monitoring - Tailored feedback |
| Gund et al. (2013)  *Sweden* | Randomized controlled trial | Investigating whether the use of conferencing or a web application improves parents’ satisfaction in taking care of a premature infant at home and decreases the need of home visits;  Examining nurses’ attitudes regarding the use of these tools | LOW-RISK neonatal  Families  (*N* = 34) | Care@  Distance Web App;  Skype video conferencing; Tailored feedback; On demand information and resources | - Targeted client communication - On-demand information services | N/A | - On-demand information and resources - Remote communication - Tailored feedback |
| Halili et al. (2018)  *Canada* | Qualitative descriptive | Exploring women’s attitudes towards the SmartMoms lifestyle App | HIGH-RISK antenatal  Pregnant women at risk for  unhealthy GWG  (*N* = 14) | Smartphone App;  Fitbit activity tracker; Resource for mental health & mindfulness techniques | - Targeted client communication - Personal health tracking - On-demand information services | N/A | - Decision support - Goal setting - On-demand information and resources - Self-monitoring - Tailored feedback |
| Hantsoo et al. (2018)  *USA* | Randomized controlled trial | Testing whether a mood tracking and alert (MTA) mobile App improved mental health care delivery | HIGH-RISK antenatal  Pregnant women with low Socioeconomic status  (*N* = 72) | App:  Digital Patient Portal (allows email communication with providers), self-monitoring of mood, tailored-feedback based on mood data | - Targeted client communication | Patient engagement and care satisfaction questionnaire items | - Remote communication - Tailored feedback |
| Harris et al. (2015)  *USA* | Two group randomized pilot design | Evaluating a 6-week long web-based contingency management program (CM) and a phone-delivered cessation counseling program (Smoking Cessation for Healthy Births [SCHB]) with pregnant smokers in rural Appalachia. | HIGH-RISK antenatal  Pregnant women =<12 GW (*N*=17) | Motiv8, an internet technology (functionality with mobile possible) used to verify breath carbon monoxide measurements paired with piCO Smokerlyzer, and web camera;  Laptops were offered if needed by participants | - Targeted client communication - Personal health tracking - On-demand information services | N/A | - On-demand information and resources - Self-monitoring - Tailored feedback |
| Hawkins et al. (2019)  *USA* | Pilot RCT  (Protocol) | (1) Establishing the feasibility and acceptability of conducting a 12-week intervention for sleep self-management among pregnant women using a wearable device  (2) Determining the feasibility of collecting data on sleep and physical activity with wearable | LOW-RISK antenatal  Pregnant women  14-24 GW  >=18 yoa  (*N* =24) | Tri-axial accelerometer wearable (Shine 2) device to monitor sleep for 12 weeks | - Targeted client communication - Personal health tracking | Proportion of days wearable was used | - Decision support - Goal setting - Self-monitoring - Tailored feedback |
| Herbec et al. (2014)  *UK* | Two-arm double-blind pilot design | Evaluating preliminarily the effectiveness and usage of a fully automated smoking cessation website | HIGH-RISK antenatal  Pregnant women (*N*=200) | Website with tailored information; online self-assessment questionnaire | - Targeted client communication - Personal health tracking - On-demand information services | Website activity through website analytics | - Goal setting - On-demand information and resources - Self-monitoring - Tailored feedback |
| Herring et al. (2019)  *USA* | Two-armed Randomized controlled trial | Testing the effectiveness of a mobile health intervention to minimize excessive GWG verses usual care | HIGH-RISK antenatal  AA Pregnant women  <22 GW <18 yoa  (*N* =262) | Digital self-monitoring program; Using iOTA weight loss approach and Behavior change goals, decision-support, tailored feedback and on-demand generic information, resources and tailored information | - Targeted client communication - Client to client communication - Personal health tracking - On-demand information services | N/A | - Decision support - Goal setting - On-demand information and resources - Peer support - Self-monitoring - Tailored feedback |
| Himes et al. (2017)  *USA* | Randomized controlled feasibility trial | (1) Refining and assessing the usability of Healthy Beyond Pregnancy;  (2) Assessing the feasibility of a randomized controlled trial of the intervention | LOW-RISK postpartum  Women  6-72h postpartum  18-50 yoa  (*N* = 30) | Web-based resource:  SMS messaging, decision-support, and tailored feedback | - Targeted client communication | Adherence to postpartum visits & and long-acting reversible contraception | - Decision support - Tailored feedback |
| Hirshberg et al. (2018)  *USA* | Randomized controlled trial | Comparing the effectiveness of text-based blood pressure monitoring to in-person visits for women with hypertensive disorders of pregnancy in the immediate postpartum period | HIGH-RISK postpartum  Postpartum women discharged from hospital with GHTN (*N* = 206) | Text messaging feedback system & remote blood pressure self-monitoring program, on-demand information and resources | - Targeted client communication - Personal health tracking - On-demand information services | Observance of frequency and volume of reported blood pressure values submitted by women | - Decision-support - On-demand information and resources - Remote Communication - Self-Monitoring - Tailored Feedback |
| Holm et al. (2019)  *Denmark* | Phase three participatory design | Exploring the in-depth parental experiences of a neonatal tele-homecare (NTH) service | LOW-RISK neonatal  Parents whose preterm infants were admitted to NICU  (*N* = 49) | Tele-Health App;  Weigh scale;  Digital parent assessment log | - Targeted client communication - Personal health tracking - On-demand information services | N/A | - Decision support - Digital log keeping - On-demand information and resources - On-demand tailored information - Remote communication - Self-monitoring - Tailored feedback |
| Isetta et al. (2013)  *Spain* | Retrospective cohort study | Evaluating the cost-effectiveness of a new Internet-based system for monitoring low-risk newborns after discharge compared to the standard hospital-based follow-up, with specific attention to prevention of emergency department (ED) visits in the first month of life | LOW-RISK neonatal  Families  (*N* = 230) | Babies at home App: Baby care & breastfeeding information;  Two-way communication;  Digital parent assessment journal and note keeping log;  Nurse online assessment with questionnaire; Decision support; Tailored-feedback | - Targeted client communication - Personal health tracking - On-demand information services | N/A | - Digital log keeping - Decision support - On-demand information and resources - Remote communication - Self-monitoring - Tailored feedback |
| Janssen et al.  (2021)  *USA* | Multicenter implementation study | assess the scalability and implementation of a previously validated, remote, postpartum BP monitoring program. | HIGH-RISK  postpartum  Postpartum Women  (*N*=199) | Way to Health platform for electronic medical record and text-based surveillance of Blood Pressure  BP cuff (Omron series 3) | - Targeted client communication - Personal health tracking - On-demand information services | User engagement was deﬁned as the submission of at least 1 BP measurement via text message through the program in the 10 days immediately following discharge | - Decision-support - On-demand information and resources - Remote Communication - Self-Monitoring - Tailored Feedback |
| Jefferson et al. (2019)  *USA* | User-centered design | Engaging mothers in the development of the Mother's Milk Connection App | LOW-RISK postpartum  Postpartum women  >18 yoa  (*N* = 10) | Mother’s Milk Connection App:  Peer support; Asynchronous chat with  health care provider; Zoom meetings; informational videos & other breastfeeding resources;  Digital breastfeeding log with trend visualizations | - Targeted client communication - Client to client communication - Personal health tracking | N/A | - Decision support - Goal setting - Peer support - Remote communication - Self-monitoring - Tailored feedback |
| Ke et al.  (2019)  *Canada* | Exploratory qualitative study | Involving patients and anesthesiologists in designing a mobile application to enhance the perioperative care of elective CD patients | LOW-RISK  perinatal  Pregnant women undergoing an elective CD  (*N*=15)  Obstetric anesthesiology staff  (*N*=9) | The C-Care© mobile application for the anesthesiology pre-postoperative care of elective CD patients | - Targeted client communication - Personal health tracking - On-demand information services | Volume of interactions with the mobile application | - Digital log keeping - On-demand information and resources - Tailored feedback |
| Ke et al.  (2019)  *Canada* | Prospective cohort study | Obtaining feedback on patient engagement with C-Care© Application | LOW-RISK  perinatal  Pregnant women undergoing an elective CD  (*N*= 36) | The C-Care© mobile application for the anesthesiology pre-postoperative care of elective CD patients | - Targeted client communication - Personal health tracking - On-demand information services | Number and  percentage of self-monitoring questionnaires completed, and the number of total visits to the mobile application per participant within 30 postoperative days.  Evaluated multiple user uptake and behavioral characteristics that are of interest for further development and implementation: time of the day participants visited, time of the day participants ﬁlled out the questionnaire, number of visits on each postoperative day, duration (number of days starting from delivery date until the last day of visit to C-Care) of interaction within 30 postoperative days, the number of information topics viewed, ranking of the most-viewed topics, ranking of the most-completed questionnaire by number of postoperative days, and incidence of self-check symptoms entered by the participant. | - Digital log keeping - Tailored feedback - On-demand information and resources |
| Kennelly et al. (2016)  *UK* | Two-armed Randomized controlled trial (Protocol) | Assessing the impact of a ‘healthy lifestyle package’ with smartphone technology as support, compared with usual care on the incidence of GDM | HIGH-RISK antenatal  Pregnant women  >29 GW  Goal  (*N* = 506) | Intervention delivered through one face-to-face session and regular contact through email and use of App | - Targeted client communication - Personal health tracking - On-demand information services | A log of lifestyle behaviors recorded by participants | - On-demand information and resources - Remote communication - Self-monitoring - Tailored Feedback |
| Kennelly et al.  (2018)  *UK* | Two-armed Randomized controlled trial | Assessing the impact of a ‘healthy lifestyle package’ with smartphone technology as support, compared with usual care on the incidence of GDM | HIGH-RISK antenatal  Pregnant women  >29 GW  (*N* = 565) | Intervention delivered through one face-to-face session and regular contact through email and use of App | - Targeted client communication - Personal health tracking - On-demand information services | A log of lifestyle behaviors recorded by participants | - On-demand information and resources - Remote communication - Self-monitoring - Tailored Feedback |
| Krishnamurti et al. (2017)  *USA* | Proof of concept pilot | Developing an engaging, usable smartphone app that communicates personalized pregnancy risk and gathers risk data, with the goal of decreasing preterm birth rates in a typically hard-to-engage patient population | HIGH-RISK antenatal  Pregnant women  (*N*= 16; 5 women from each trimester) | My Healthy  Pregnancy App: connected devices and resources (ie. Weigh scale, informational resources), decision support, and tailored-feedback | - Targeted client communication - Personal health tracking - On-demand information services | N/A | - Decision Support - On-demand information and resources - Self-monitoring - Tailored Feedback |
| Ledford et al. (2015)  *USA* | Randomized controlled pilot | Testing the effectiveness of a mobile app as a replacement for a spiral notebook guide as a patient education and engagement tool in the prenatal clinical setting | LOW-RISK antenatal  Pregnant women  10-12 GW (*N*=173) | Mobile App serving as a journal for women to  record parameter data they collect at home or in the clinic  and their experiences in pregnancy; education resource on matters relating to healthy pregnancy | - Personal health tracking - On-demand information services | N/A | - Digital log keeping - On-demand information and resources - Self-monitoring |
| Ledford et al.  (2017)  *USA* | Randomized controlled trial | Testing the effectiveness of a mobile app as a replacement for a spiral notebook guide as a patient education and engagement tool in the prenatal clinical setting | LOW-RISK antenatal  Pregnant women  10-12 GW (*N*=205) | Mobile App serving as a journal for women to  record parameter data they collect at home or in the clinic  and their experiences in pregnancy and as an education resource on matters relating to health pregnancy | - Personal health tracking - On-demand information services | N/A | - Digital log keeping - On-demand information and resources - Self-monitoring |
| Lee et al.  (2016)  *USA* | Design report (conference report) | Developing a program to help pregnant women find their nausea and vomiting patterns more quickly and avoid triggers | HIGH-RISK antenatal  Pregnant women in 2^nd^ and 3^rd^ trimester (*N*=5)  HCPs  (*N* = 2) | Dot-it App:  Daily recorder (wrist band);  Digital planner & time management tool, Decision support; Tailored feedback; On-demand resource for symptom management | - Targeted client communication - Client to client communication - Personal health tracking - On-demand information services | N/A | - Decision support - On-demand information and resources - Partnered paired device - Self-monitoring - Tailored feedback |
| Marko et al. (2016)  *USA* | Prospective observational study | Determining the  feasibility of using digital health tools to manage prenatal care | LOW-RISK antenatal  Pregnant women  1^st^ trimester  (*N* = 6) | Babyscripts App: connected to a wireless weight scale and sphygmomanometer;  Delivered evidence-based educational information related to prenatal care at gestational age-specific times during pregnancy in the form of a to-do list | - Targeted client communication - Personal health tracking - On-demand information services | Patient satisfaction (patient provider relationship-engagement-patient education);  Engagement through usage of App and self-monitoring | - Decision support - On-demand information and resources - On-demand tailored information - Self-monitoring - Tailored feedback |
| Marko et al.  (2019)  *USA* | Prospective controlled trial | Testing the effectiveness of a mobile prenatal care app to facilitate a reduced in-person visit schedule for low-risk pregnancies while maintaining patient and provider satisfaction | LOW-RISK antenatal  Pregnant women  =>13 GW  18-40 yoa  (*N* = 88) | Babyscripts App: connected to a  wireless weight scale and sphygmomanometer;  Delivered evidence-based educational information related to prenatal care at gestational age-specific times during pregnancy in the form of a to-do list | - Targeted client communication - Personal health tracking - On-demand information services | Number of in-person prenatal visits  Patient satisfaction (patient provider relationship-engagement-patient education) | - Decision support - On-demand information and resources - On-demand tailored information - Self-monitoring - Tailored feedback |
| Muuraiskangas et al. (2016)  *Finland* | Field study report of a feasibility RCT | Specifically, the aim was to study the barriers of using the  application and the experienced benefits of the application among pregnant women." | LOW-RISK antenatal  Pregnant women  16-18 GW and >18 yoa  (*N* = 53)  PHN  (N = 52) | Oiva App with activities that educate and train women  in mindfulness  self-monitoring with diary notes and tailored  feedback based on stated preferences and progress towards goals (widget information) | - Targeted client communication - Personal health tracking - On-demand information services | Usage logs were collected from the study phones at the end of the study period | - Digital log keeping - On-demand information and resources - Self-monitoring - Tailored feedback |
| Naughton et al. (2012)  *UK* | RCT | To assess feasibility and acceptability of a tailored self-help smoking cessation intervention for pregnant smokers (MiQuit). | HIGH-RISK antenatal  Pregnant women 21 GW, =>16 years old (*N* = 207) | MiQuit SMS based self-help intervention based on principles of Social Cognitive Theory:  Remote communication; Goal setting; Self-monitoring; On demand tailored information | - Targeted client communication - Personal health tracking - On-demand information services | N/A | - Goal setting - On-demand tailored information - Remote communication - Self-monitoring |
| Naughton et al. (2013)  *UK* | Qualitative Study | Explore the attitudes of women with experience of prenatal smoking towards receiving pregnancy-related smoking cessation support by text message. | HIGH-RISK antenatal  Pregnant women (*N* = 33) | MiQuit SMS based self-help intervention based on principles of Social Cognitive Theory. | - Targeted client communication - Personal health tracking - On-demand information services | N/A | - Goal setting - On-demand tailored information - Remote communication - Self-monitoring |
| Naughton et al. (2017)  *UK* | Multicenter, parallel-group, single blind, individual RCT | To estimate the effectiveness of pregnancy smoking cessation support delivered by SMS text message and key parameters needed to plan a definitive trial. | HIGH-RISK antenatal  Pregnant women <25 GW, =>16 years old (*N* = 407) | MiQuit SMS based self-help intervention based on principles of Social Cognitive Theory. | - Targeted client communication - Personal health tracking - On-demand information services | N/A | - Goal setting - On-demand tailored information - Remote communication - Self-monitoring |
| O’Brien et al.  (2013)  *UK* | Qualitative study | To gain insight into women’s experiences and preferences for induction in the home as a part of a trial investigating the feasibility and acceptability of outpatient induction of labour with remote monitoring | LOW-RISK peripartum  Peripartum women (*N* = 15) | Wireless monitoring device relays fetal ECG, uterine activity and maternal heart rate in real time to the hospital, and there reviewed by midwifery staff.  Remote communication; Decision support. | - Targeted client communication - Personal health tracking | Self-completed diaries kept by peripartum women | - Decision support - Remote communication - Self-monitoring |
| Payakachat et al.  (2020)  *USA* | Pilot, non- randomized cohort study | (1) Exploring perceptions and attitudes of postpartum women toward mHealth; (2) Identifying facilitators and barriers of using mHealth; and (3) Documenting views toward call center communication for integrating mHealth into possible standard of care. | HIGH-RISK postpartum  Postpartum women discharged from hospital with preeclampsia  =>18 years old (*N* = 48) | mHealth devices to be used for self-monitoring blood pressure, weight, pulse, and oxygen saturation;  The nurse call center monitored device readings and contacted participants as needed | - Targeted client communication - Personal health tracking | Divided groups into user and non-user groups for comparison | - Remote Communication - Self-monitoring |
| Platonos et al. (2018)  *UK* | Program report | Creating complex experience co-designed training material with the use of Family Integrated Care App | HIGH-RISK neonatal  Families  (*N* not reported) | Family Integrated Care (FICare) App:  Digital journal for parental tasks and close time spent with infants;  Data collected in journal can be used in FICare peer and professional coaching | - Personal health tracking - On-demand information services | N/A | - Digital log keeping - On-demand information and resources |
| Rhoads et al. (2016)  *USA* | Pilot, non- randomized cohort study | Identifying and examining the potential factors that influenced use of m-health technology and adherence to monitoring symptoms related to preeclampsia in postpartum women | HIGH-RISK postpartum  Postpartum women discharged from hospital with preeclampsia  =>18 years old (*N* = 48) | mHealth devices to be used for self-monitoring blood pressure, weight, pulse, and oxygen saturation;  The nurse call center monitored device readings and contacted participants as needed | - Targeted client communication - Personal health tracking | Treatment adherence;  Divided groups into user and non-user groups for comparison | - Remote Communication - Self-monitoring |
| Ridgeway et al. (2015)  *USA* | Randomized controlled trial- mixed methods (Protocol) | Determining the effectiveness and feasibility of a new prenatal care model (OB Nest) that enhances a reduced visit model by adding virtual connections that improve continuity of care and patient-directed access to care | LOW-RISK antenatal  Pregnant women RCT:  Goal  (*N* = 300)  Qualitative Methods:  Focus groups-Pregnant women  Goal  (*N=* 40)  Nurses  Goal  (*N* = 7)  Midwives  Goal  (*N* = 19)  Clinical assistants  Goal  (*N* = 10)    Individual interviews:  Physicians  Goal  (*N* = 6) | 6 virtual visits (phone/online communicat-ion  with nurses);  Fetal Doppler and sphygmomano-meter home  monitoring devices; Online community of pregnant women; Medical records for submitting parameter data to nurses if they wish to use (can also send  data by phone) | - Targeted client communication - Client to client communication - Personal health tracking - On-demand information services | Adherence measurement (healthcare utilization) | - Decision support - On-demand information and resources - On-demand tailored information - Peer support - Remote communication - Self-monitoring - Tailored feedback |
| Salonen et al. (2010)  *Finland* | Quasi-experimental design with a non-equivalent control group | Evaluating the effectiveness of an internet-based intervention to support mothers’ and fathers’ parenting satisfaction and parenting self-efficacy | LOW-RISK neonatal  Families  (*N* = 1300) | Website and patient portal:  Remote communication;  Peer support;  On-demand tailored information | - Targeted client communication - Client to client communication - On-demand information services | N/A | - On-demand tailored information - Peer support - Remote communication |
| Shorey et al. (2016)  *Canada* | Randomized controlled trial  (Protocol) | Describing protocol for evaluating the effectiveness of the Home-but-not-alone educational program delivered via App in improving parenting outcomes | LOW-RISK neonatal  Families  Goal  (*N* = 118) | Home-but-not-alone App:  discussion forum that enabled parents to relay photographs or messages and have their queries and concerns addressed by a midwife once within 24 hours;  Extensive information resource comprising audios, videos, and PDF documents on new born, maternal, and paternal care; Daily notifications received by the parents regarding their babies’ important milestones & needs; Asynchronous mode of communication was used by the midwife to answer parental inquiries once a day; Decision support; Peer support; On demand tailored information, generic information and resources | - Targeted client communication - Client to client communication - On-demand information services | N/A | - Decision support - On-demand information and resources - On-demand tailored information - Peer support - Remote communication - Tailored feedback |
| Shorey et al. (2017)  *Canada* | Randomized controlled trial | Evaluating the effectiveness of the Home-but-not-alone educational program delivered via App in improving parenting outcomes | LOW-RISK neonatal  Parents  (*N* = 250) | Home-but-not-alone App:  discussion forum that enabled parents to relay photographs or messages and have their queries and concerns addressed by a midwife once within 24 hours;  Extensive information resource comprising audios, videos, and PDF documents on new born, maternal, and paternal care; Daily notifications received by the parents regarding their babies’ important milestones & needs; Asynchronous mode of communication was used by the midwife to answer parental inquiries once a day; Decision support; Peer support; On demand tailored information, generic information and resources | - Targeted client communication - Client to client communication - On-demand information services | N/A | - Decision support - On-demand information and resources - On-demand tailored information - Peer support - Remote communication - Tailored feedback |
| Shorey et al. (2018)  *Canada* | Descriptive qualitative study | Exploring the views of parents of newborns with regard to the content and delivery of a mobile health (mHealth) app–based postnatal educational program | LOW-RISK neonatal  Parents  (*N* = 17) | Home-but-not-alone App:  discussion forum that enabled parents to relay photographs or messages and have their queries and concerns addressed by a midwife once within 24 hours;  Extensive information resource comprising audios, videos, and PDF documents on new born, maternal, and paternal care; Daily notifications received by the parents regarding their babies’ important milestones & needs; Asynchronous mode of communication was used by the midwife to answer parental inquiries once a day; Decision support; Peer support; On demand tailored information, generic information and resources | - Targeted client communication - Client to client communication - On-demand information services | N/A | - Decision support - On-demand information and resources - On-demand tailored information - Peer support - Remote communication - Tailored feedback |
| Soltani et al.  (2015)  *UK* | Feasibility study | Exploring the appropriateness of a text messaging based complex intervention for promoting healthy GWG during pregnancy | HIGH-RISK antenatal  Pregnant women  8-10 GW  (*N* = 14) | Text message program adjunct to goal setting program delivered by midwives; On demand information and resources; & self-monitoring | - Personal health tracking - On-demand information services | N/A | - Goal setting - On-demand information and resources - Self-monitoring |
| Spargo et al.  (2018)  *New Zealand* | Program report | Reporting on the development of the ‘Babble’ application | LOW-RISK/HIGH-RISK  neonatal  Sets of parents  (*N* = 7) | Babble Family Neonatal Application with information on-demand and a possibility to add journal entries about their memories in the NICU | - Personal health tracking - On-demand information services | N/A | - Digital log keeping - On-demand information and resources |
| Strand et al.  (2021)  *Sweden* | Participatory design guided by the Framework for Complex Interventions in Health Care | Developing an eHealth device supporting the transition from  hospital to home for parents with a preterm- born child in Sweden by the use of participatory design | HIGH-RISK  neonatal  Initial phase Only HCPs  (*N* = not reported)  Second phase  Families who were staying in the NICU  (*N* = 3)  Third phase  Families  (in NICU)  (*N* = 3)  Families  (at home)  (*N* = 5) | The eHealth program includes the following through an Android tablet device:  • Professionals: Track the patient's medical data. Three initial data types: weight, oxygenation and food intake.  • Professionals: View patient in live video stream using video call.  • Parents: Direct communication with known personnel, text messages and video calls.  • Parents: Secure method of sending photographs.  • Parents: Track weight development on graph, easily identify trends. | - Targeted client communication - Personal health tracking | N/A | - Remote communication - Self-monitoring |
| Tobah et al. (2019)  *USA* | Randomized controlled trial | Evaluating the acceptability and effectiveness of OB Nest, a reduced-frequency prenatal care model enhanced with remote home monitoring devices and nursing support | LOW-RISK antenatal  Pregnant women  <13 GW  18-36 yoa (*N* = 300) | 6 virtual visits (phone/online communicat-ion with nurses);  Fetal Doppler and sphygmomano-meter home  monitoring devices; Online community of pregnant women; Medical records for submitting parameter data to nurses if they wish to use (can also send  data by phone) | - Targeted client communication - Client to client communication - Personal health tracking - On-demand information services | N/A | - Decision support - On-demand information and resources - On-demand tailored information - Peer support - Remote communication - Self-monitoring - Tailored feedback |
| Tommasone et al. (2016)  *Italy* | Design and Implementat-ion report | Designing and implementing a mobile App Mammastyle-Gravidanza Fisiologica | LOW-RISK antenatal  Pregnant women  =12 GW  18-39 yoa  (*N* = 6)  Midwives & Obstetric-ians  (*N* = 6) | Mammastyle App and website: monitor daily pregnant women’s lifestyles, evaluating the diet, the physical activity and the hydration, to promote the adoption of healthy habits in order to prevent overweight and obesity pregnancy, limiting the gestational weight gain as suggested by IOM (Institute of Medicine); Self-monitoring; on demand tailored and generic information; on demand resources; Digital patient logs for note keeping | - Targeted client communication - Personal health tracking - On-demand information services | N/A | - Digital log keeping - On-demand information and resources - On-demand tailored information - Self-monitoring |
| Triebwasser et al.  (2020)  *USA* | Retrospective cohort study | Comparing BP ascertainment outcomes between  trial participants and women clinically enrolled after implementation of Heart Safe Motherhood in a new institutional setting | HIGH-RISK  postpartum  Postpartum women  (*N* = 333) | Way to Health platform for electronic medical record and text-based surveillance of Blood Pressure  BP cuff (Omron series 3) | - Targeted client communication - Personal health tracking - On-demand information services | BP ascertainment, defined as the  percentage of patients in which a single BP was obtained in the first 10 days following discharge. | - Decision-support - On-demand information and resources - Remote Communication - Self-Monitoring - Tailored Feedback |
| Valencia et al.  (2020)  *USA* | Pilot evaluation and design | Presenting the design of the MoMba Live Long , mobile, breath CO meter and a pilot evaluation of the feasibility of the system as a smoking assessment tool during and after pregnancy | HIGH-RISK  antenatal  Pregnant women  (*N*= 10) | Smoking cessation remote monitoring and incentive program, using a the MoMba Live Long App and mobile piCo+ monitor. | - Targeted client communication - Personal health tracking - On-demand information services | Response rate to breath test notifications | - Digital log keeping - Goal setting - On-demand information and resources - Self-monitoring - Tailored feedback |
| van der Wulp et al. (2014)  *The Netherlands* | Cluster randomized trial | Testing the effectiveness of two different brief interventions to reduce prenatal alcohol use, a health counseling and computer-tailored intervention, in comparison with usual care | HIGH-RISK antenatal  Pregnant women =>18 yoa 12 GW | Computer-tailored feedback program for reducing alcohol use; On demand tailored information; On demand information and resources; Goal setting | - Targeted client communication - Personal health tracking - On-demand information services | N/A | - Goal setting - On-demand information and resources - On-demand tailored information - Self-monitoring |
| Whitemore et al. (2019)  *UK* | Randomized controlled trial (Protocol) | Evaluating and demonstrating whether or not MiQuit program is efficacious for smoking cessation in pregnancy | HIGH-RISK antenatal  Pregnant women  <25 GW =>16 yoa (*N* =692) | MiQuit SMS based self-help intervention based on principles of Social Cognitive Theory | - Targeted client communication - Personal health tracking - On-demand information services | Survey data collection at four time points (one pre-trial) | - Goal setting - On-demand tailored information - Self-monitoring - Remote communication |
| Wierckx et al. (2014)  *The Netherlands* | User design conference report | Evaluating design and development of Babywijzer App for perinatal care support | LOW-RISK antenatal  First interviews: Pregnant women  (*N* = 13)  Obstetricians  (*N* = 3)  Proto-type testing:  Pregnant women  (*N* = 11)  Obstetricians  (*N* = 1) | Babywijzer App:  (1) A complete personal health record system;  (2) Women can monitor their own health records (weight gain and blood pressure);  (3) A health meter gives them an indication of their eating habits e.g. enough fruits and vegetables per day;  (4) A push notification system for accomplishing different tasks; (5) An alarm system for warnings (e.g. high blood pressure, abnormal weight gain, bad eating habits, etc.);  (6) A journal module;  (7) A module to send health records to obstetricians; 8) A section on social rules that prevent women from risky behaviors;  (9) A module to connect with other pregnant women; Tailored feedback; & Self-monitoring | - Targeted client communication - Client to client communication - Personal health tracking | N/A | - Decision support - Goal setting - Peer support - Remote communication - Self-monitoring - Tailored feedback |
| Willcox et al. (2015)  *Australia* | Two-armed Randomized controlled trial (Protocol) | Testing the feasibility of an mHealth intervention promoting healthy nutrition, physical activity, and GWG | HIGH-RISK antenatal  Pregnant women  >18 yoa  10-17^+6^ GW  BMI pre-  pregnancy >25kg/m^2^ (*N* = 100) | Multi-dimensional interventions include tailored text messages; Access to a Multi-dimensional interventions include tailored text messages; Access to a responsive information website;  Video messages; Chat room interaction via Facebook® | - Targeted client communication - Client to client communication - Personal health tracking - On-demand information services | Metrics of use of each component: for example, web analytics for page views, time on individual pages, duration of visits, intensity of use, replies to messages, visits to website, views of and contributions to use of Facebook® chat forum | - Goal setting - On-demand information and resources - Peer support - Self-monitoring - Tailored feedback |
| Willcox et al.  (2017)  *Australia* | Pilot RCT | To determine the feasibility and effectiveness of an mHealth intervention promoting health diet, physical activity and GWG in pregnancy. | HIGH-RISK antenatal  Pregnant women  >18 yoa  10-17^+6^ GW  BMI pre-Pregnancy >25kg/m^2^ (*N* = 91) | Responsive information website;  Video messages; Chat room interaction via Facebook® | - Targeted client communication - Client to client communication - Personal health tracking - On-demand information services | Metrics of use of each component: for example, web analytics for page views, time on individual pages, duration of visits, intensity of use, replies to messages, visits to website, views of and contributions to use of Facebook® chat forum | - Goal setting - On-demand information and resources - Peer support - Self-monitoring - Tailored feedback |
| Yee et al.  (2021)  *USA* | Prospective qualitative study | Eliciting feedback from patients and healthcare providers regarding usability of the SweetMama prototype | HIGH-RISK  antenatal  Patients with T2DM or GDM | The SweetMama application is a goal setting and information resource in the form of an educational and motivational mHealth tool for pregnancy with diabetes | - Targeted client communication - On-demand information services | N/A | - Goal setting - On-demand information and resources - On-demand tailored information - Remote communication - Tailored feedback |

Footnotes: N/A= Not applicable; AA=African American; CD= Caesarean Delivery; GDM=Gestational diabetes mellitus; GHTN=Gestational hypertension; GP=General practitioner; GW=Gestational weeks; GWG=Gestational weight gain; NICU=Neonatal Intensive Care; PCC=Patient Centered Care; PHN=Public health nurses; SMS=Short message service; T2DM=Type 2 gestational diabetes mellitus; yoa=Years of age; iOTA=interactive obesity treatment approach

# S2: Reference List of Included Sources

Abbass-Dick, J., Xie, F., Koroluk, J., Alcock Brillinger, S., Huizinga, J., Newport, A., Goodman, W. M., & Dennis, C.-L. (2017). The Development and piloting of an eHealth breastfeeding resource targeting fathers and partners as co-parents. *Midwifery*, *50*, 139–147. https://doi.org/10.1016/j.midw.2017.04.004

Abroms, L. C., Johnson, P. R., Leavitt, L. E., Cleary, S. D., Bushar, J., Brandon, T. H., & Chiang, S. C. (2017). A Randomized Trial of Text Messaging for Smoking Cessation in Pregnant Women. *American Journal of Preventive Medicine*, *53*(6), 781–790. https://doi.org/10.1016/j.amepre.2017.08.002

Banerjee, J., Aloysius, A., Mitchell, K., Silva, I., Rallis, D., Godambe, S. V., & Deierl, A. (2020). Improving infant outcomes through implementation of a family integrated care bundle including a parent supporting mobile application. *Archives of Disease in Childhood - Fetal and Neonatal Edition*, *105*(2), 172–177. https://doi.org/10.1136/archdischild-2018-316435

Baron, A. M., Ridgeway, J. L., Finnie, D. M., Stirn, S. L., Morris, M. A., Branda, M. E., Inselman, J. W., & Baker, C. A. (2018). Increasing the Connectivity and Autonomy of RNs with Low-Risk Obstetric Patients: Findings of a study exploring the use of a new prenatal care model. *AJN American Journal of Nursing*, *118*(1), 48–55. cin20. https://doi.org/10.1097/01.NAJ.0000529715.93343.b0

Baruth, M., Schlaff, R. A., Deere, S., Walker, J. L., Dressler, B. L., Wagner, S. F., Boggs, A., & Simon, H. A. (2019). The Feasibility and Efficacy of a Behavioral Intervention to Promote Appropriate Gestational Weight Gain. *Maternal & Child Health Journal*, *23*(12), 1604–1612. cin20. https://doi.org/10.1007/s10995-019-02812-6

Bower, D. J., Barry, N., Reid, M., & Norrie, J. (2005). Designing and implementing E-health applications in the UK’s National Health Service. *Journal of Health Communication*, *10*(8), 733–750. https://doi.org/10.1080/10810730500326732

Carrilho, J. M., Oliveira, I. J. R., Santos, D., Osanan, G. C., Cruz-Correia, R. J., & Reis, Z. S. N. (2019). Pregnant Users’ Perceptions of the Birth Plan Interface in the “My Prenatal Care” App: Observational Validation Study. *JMIR Formative Research*, *3*(1), e11374. https://doi.org/10.2196/11374

Choi, J., Lee, J. hyeon, Vittinghoff, E., & Fukuoka, Y. (2015). mHealth Physical Activity Intervention: A Randomized Pilot Study in Physically Inactive Pregnant Women. *Maternal and Child Health Journal*, *20*(5), 1091–1101. https://doi.org/10.1007/s10995-015-1895-7

Cramer, M. E., Mollard, E. K., Ford, A. L., Kupzyk, K. A., & Wilson, F. A. (2018). The feasibility and promise of mobile technology with community health worker reinforcement to reduce rural preterm birth. *Public Health Nursing*, *35*(6), 508–516. cin20. https://doi.org/10.1111/phn.12543

Dalton, J. A., Rodger, D., Wilmore, M., Humphreys, S., Skuse, A., Roberts, C. T., & Clifton, V. L. (2018). The Health-e Babies App for antenatal education: Feasibility for socially disadvantaged women. *Plos One*, *13*(5), e0194337. https://doi.org/10.1371/journal.pone.0194337

Danbjørg, D. B., Wagner, L., Kristensen, B. R., & Clemensen, J. (2015). Intervention among new parents followed up by an interview study exploring their experiences of telemedicine after early postnatal discharge. *Midwifery*, *31*(6), 574–581. https://doi.org/10.1016/j.midw.2015.02.007

Danbjørg, D.B., Wagner, L., & Clemensen, J. (2014). Designing, Developing, and Testing an App for Parents Being Discharged Early Postnatally. *Journal for Nurse Practitioners*, *10*(10), 794–802. cin20. https://doi.org/10.1016/j.nurpra.2014.07.023

Davis, D., Davey, R., Williams, L. T., Foureur, M., Nohr, E., Knight-Agarwal, C., Lawlis, T., Oats, J., Skouteris, H., & Fuller-Tyszkiewicz, M. (2018). Optimizing Gestational Weight Gain with the Eating4Two Smartphone App: Protocol for a Randomized Controlled Trial. *JMIR Research Protocols*, *7*(5), e146. https://doi.org/10.2196/resprot.9920

de Mooij, M. J. M., Hodny, R. L., O’Neil, D. A., Gardner, M. R., Beaver, M., Brown, A. T., Barry, B. A., Ross, L. M., Jasik, A. J., Nesbitt, K. M., Sobolewski, S. M., Skinner, S. M., Chaudhry, R., Brost, B. C., Gostout, B. S., & Harms, R. W. (2018). OB Nest: Reimagining Low-Risk Prenatal Care. *Mayo Clinic Proceedings*, *93*(4), 458–466. https://doi.org/10.1016/j.mayocp.2018.01.022

Demirci, J. R., Suffoletto, B., Doman, J., Glasser, M., Chang, J. C., Sereika, S. M., & Bogen, D. L. (2020). The Development and Evaluation of a Text Message Program to Prevent Perceived Insufficient Milk Among First-Time Mothers: Retrospective Analysis of a Randomized Controlled Trial. *JMIR MHealth and UHealth*, *8*(4), e17328. https://doi.org/10.2196/17328

Demment, M. M., Graham, M. L., & Olson, C. M. (2014). How an Online Intervention to Prevent Excessive Gestational Weight Gain Is Used and by Whom: A Randomized Controlled Process Evaluation. *Journal of Medical Internet Research*, *16*(8), e194. https://doi.org/10.2196/jmir.3483

Doherty, K., Barry, M., Marcano-Belisario, J., Arnaud, B., Morrison, C., Car, J., & Doherty, G. (2018). A Mobile App for the Self-Report of Psychological Well-Being During

Pregnancy (BrightSelf): Qualitative Design Study. *JMIR Mental Health*, *5*(4), e10007. https://doi.org/10.2196/10007

Doherty, K., Barry, M., Belisario, J. M., Morrison, C., Car, J., & Doherty, G. (2020). Personal information and public health: Design tensions in sharing and monitoring wellbeing in pregnancy. *International Journal of Human-Computer Studies*, *135*, 102373. https://doi.org/10.1016/j.ijhcs.2019.102373

Doherty, K., Marcano-Belisario, J., Cohn, M., Mastellos, N., Morrison, C., Car, J., & Doherty, G. (2019). Engagement with Mental Health Screening on Mobile Devices: Results from an Antenatal Feasibility Study. *Proceedings of the 2019 CHI Conference on Human Factors in Computing Systems*, 1–15. https://doi.org/10.1145/3290605.3300416

Dougall, G., Franssen, M., Tucker, K. L., Yu, L.-M., Hinton, L., Rivero-Arias, O., Abel, L., Allen, J., Band, R. J., Chisholm, A., Crawford, C., Green, M., Greenfield, S.,

Hodgkinson, J., Leeson, P., McCourt, C., MacKillop, L., Nickless, A., Sandall, J., … McManus, R. J. (2020). Blood pressure monitoring in high-risk

pregnancy to improve the detection and monitoring of hypertension (the BUMP 1 and 2 trials): Protocol for two linked randomised controlled trials.

*BMJ Open*, *10*(1), e034593. https://doi.org/10.1136/bmjopen-2019-034593

Fontein-Kuipers, J. A. C. A., & Vries, R. G. de. (2016). *WazzUp mama?!: The development of an intervention to prevent and reduce maternal distress during pregnancy*. DOI 10.1007/s00737-016-0614-8

Franck, L. S., Kriz, R. M., Bisgaard, R., Cormier, D. M., Joe, P., Miller, P. S., Kim, J. H., Lin, C., & Sun, Y. (2019). Comparison of family centered care with family integrated care and mobile technology (mFICare) on preterm infant and family outcomes: A multi-site quasi-experimental clinical trial protocol. *BMC Pediatrics*, *19*(1), 469. https://doi.org/10.1186/s12887-019-1838-3

Frize, M., Bariciak, E., Dunn, S., Weyand, S., Gilchrist, J., & Tozer, S. (2011). *Combined physician-parent decision support tool for the neonatal intensive care unit*. MeMeA 2011 - 2011 IEEE International Symposium on Medical Measurements and Applications, Proceedings. Scopus. https://doi.org/10.1109/MeMeA.2011.5966652

Frize, M., Bariciak, E., & Gilchrist, J. (2013). PPADS: Physician-PArent Decision-Support for neonatal intensive care. In *MEDINFO 2013* (pp. 23-27). IOS Press. 10.3233/978-1-61499-289-9-23

Garfield, C. F., Lee, Y. S., Kim, H. N., Rutsohn, J., Kahn, J. Y., Mustanski, B., & Mohr, D. C. (2016). Supporting parents of premature infants transitioning from the NICU to home: A pilot randomized control trial of a smartphone application. *Internet Interventions*, *4*, 131–137. https://doi.org/10.1016/j.invent.2016.05.004

Garne Holm, K., Brødsgaard, A., Zachariassen, G., Smith, A. C., & Clemensen, J. (2017). Participatory design methods for the development of a clinical telehealth service for neonatal homecare. *SAGE Open Medicine*, *5*, 205031211773125. https://doi.org/10.1177/2050312117731252

Garne, K., Brodsgaard, A., Zachariassen, G., & Clemensen, J. (2016). Telemedicine in Neonatal Home Care: Identifying Parental Needs Through Participatory Design. *JMIR Research Protocols*, *5*(3), 274–281. https://doi.org/10.2196/resprot.5467

Gibson, C., Ross, K., Williams, M., & de Vries, N. (2021). The Experiences of Mothers in a Neonatal Unit and Their Use of the Babble App. *SAGE Open*, *11*(2), 215824402110231. https://doi.org/10.1177/21582440211023170

Globus, O., Leibovitch, L., Maayan-Metzger, A., Schushan-Eisen, I., Morag, I., Mazkereth, R., Glasser, S., Kaplan, G., & Strauss, T. (2016). The use of short message services (SMS) to provide medical updating to parents in the NICU. *Journal of Perinatology*, *36*(9), 739–743. https://doi.org/10.1038/jp.2016.83

Graham, M. L., Uesugi, K. H., Niederdeppe, J., Gay, G. K., & Olson, C. M. (2014). *The Theory, Development, and Implementation of an e-Intervention to Prevent Excessive Gestational Weight Gain: E-Moms Roc*. *Telemedicine and e-Health*, *20*(12), 1135-1142. https://doi.org/10.1089/tmj.2013.0354

Gu, B.D., Yang, J. J., Li, J.Q., Wang, Q., & Niu, Y. (2014). Using Knowledge Management and Mhealth in High-Risk Pregnancy Care: A Case for the Floating Population in China. *2014 IEEE 38th International Computer Software and Applications Conference Workshops*, 678–683. https://doi.org/10.1109/COMPSACW.2014.114

Gund, A., Sjöqvist, B. A., Wigert, H., Hentz, E., Lindecrantz, K., & Bry, K. (2013). A randomized controlled study about the use of eHealth in the home health care of premature infants. *BMC Medical Informatics and Decision Making*, *13*(1), 22. https://doi.org/10.1186/1472-6947-13-22

Halili, L., Liu, R., Hutchinson, K. A., Semeniuk, K., Redman, L. M., & Adamo, K. B. (2018). Development and pilot evaluation of a pregnancy-specific mobile health tool: A qualitative investigation of SmartMoms Canada. *Bmc Medical Informatics and Decision Making*, *18*, 95. https://doi.org/10.1186/s12911-018-0705-8

Hantsoo, L., Criniti, S., Khan, A., Moseley, M., Kincler, N., Faherty, L. J., Epperson, C. N., & Bennett, I. M. (2018). A Mobile Application for Monitoring and Management of Depressed Mood in a Vulnerable Pregnant Population. *Psychiatric Services*, *69*(1), 104–107. https://doi.org/10.1176/appi.ps.201600582

Harris, M., & Reynolds, B. (2015). A Pilot Study of Home‐Based Smoking Cessation Programs for Rural, Appalachian, Pregnant Smokers. *Journal of Obstetric, Gynecologic & Neonatal Nursing*, *44*(2), 236–245. https://doi.org/10.1111/1552-6909.12547

Hawkins, M., Iradukunda, F., & Paterno, M. (2019). Feasibility of a Sleep Self-Management Intervention in Pregnancy Using a Personalized Health Monitoring Device: Protocol for a Pilot Randomized Controlled Trial. *JMIR Research Protocols*, *8*(5), 118–128. https://doi.org/10.2196/12455

Herbec, A., Brown, J., Tombor, I., Michie, S., & West, R. (2014). Pilot randomized controlled trial of an internet-based smoking cessation intervention for pregnant smokers (‘MumsQuit’). *Drug and Alcohol Dependence*, *140*, 130–136. https://doi.org/10.1016/j.drugalcdep.2014.04.010

Herring, S. J., Albert, J. J., Darden, N., Bailer, B., Cruice, J., Hassan, S., …& Foster, G. D. (2019). Targeting pregnancy-related weight gain to reduce disparities in obesity: Baseline results from the Healthy Babies trial. *Contemporary Clinical Trials*, *87*, 105822. https://doi.org/10.1016/j.cct.2019.105822

Himes, K. P., Donovan, H., Wang, S., Weaver, C., Grove, J. R., & Facco, F. L. (2017). Healthy Beyond Pregnancy, a Web-Based Intervention to Improve Adherence to Postpartum Care: Randomized Controlled Feasibility Trial. *JMIR Human Factors*, *4*(4), e26. https://doi.org/10.2196/humanfactors.7964

Hirshberg, A., Downes, K., & Srinivas, S. (2018). Comparing standard office-based follow-up with text-based remote monitoring in the management of postpartum hypertension: A randomised clinical trial. *BMJ Quality & Safety*, *27*(11), 871–877. cin20. https://doi.org/10.1136/bmjqs-2018-007837

Holm, K. G., Brodsgaard, A., Zachariassen, G., Smith, A. C., & Clemensen, J. (2019). Parent perspectives of neonatal tele-homecare: A qualitative study. *Journal of Telemedicine and Telecare*, *25*(4), 221–229. https://doi.org/10.1177/1357633X18765059

Isetta, V., Lopez-Agustina, C., Lopez-Bernal, E., Amat, M., Vila, M., Valls, C., Navajas, D., & Farre, R. (2013). Cost-Effectiveness of a New Internet-Based Monitoring Tool for Neonatal Post-Discharge Home Care. *Journal of Medical Internet Research*, *15*(2), e38. https://doi.org/10.2196/jmir.2361

Jefferson, U. T., Zachary, I., & Majee, W. (2019). Employing a User-Centered Design to Engage Mothers in the Development of a mHealth Breastfeeding Application. *CIN:Computers Informatics Nursing*, *37*(10), 522–531. https://doi.org/10.1097/CIN.0000000000000549

Ke, J. X. C., George, R. B., Wozney, L., & Chorney, J. L. (2019). Patient-centred perioperative mobile application in Cesarean delivery: Needs assessment and development. *Canadian Journal of Anesthesia/Journal Canadien d’anesthésie*, *66*(10), 1194–1201. https://doi.org/10.1007/s12630-019-01392-x

Ke, J. X. C., George, R. B., Wozney, L., & Munro, A. (2021). Perioperative mobile application for mothers undergoing Cesarean delivery: A prospective cohort study on patient engagement. *Canadian Journal of Anesthesia/Journal Canadien d’anesthésie*, *68*(4), 505–513. https://doi.org/10.1007/s12630-020-01907-x

Kennelly, M. A., Ainscough, K., Lindsay, K., Gibney, E., Mc Carthy, M., & McAuliffe, F. M. (2016). Pregnancy, exercise and nutrition research study with smart phone app support (Pears): Study protocol of a randomized controlled trial. *Contemporary Clinical Trials*, *46*, 92–99. https://doi.org/10.1016/j.cct.2015.11.018

Kennelly, M. A., Ainscough, K., Lindsay, K. L., O’Sullivan, E., Gibney, E. R., McCarthy, M., Segurado, R., DeVito, G., Maguire, O., Smith, T., Hatunic, M., McAuliffe, F. M., & OʼSullivan, E. (2018). Pregnancy Exercise and Nutrition With Smartphone Application Support: A Randomized Controlled Trial. *Obstetrics & Gynecology*, *131*(5), 818–826. cin20. https://doi.org/10.1097/AOG.0000000000002582

Krishnamurti, T., Davis, A. L., Wong-Parodi, G., Fischhoff, B., Sadovsky, Y., & Simhan, H. N. (2017). Development and Testing of the MyHealthyPregnancy App: A Behavioral Decision Research-Based Tool for Assessing and Communicating Pregnancy Risk. *JMIR MHealth and UHealth*, *5*(4), e42. https://doi.org/10.2196/mhealth.7036

Ledford, C. J. W., Canzona, M. R., Cafferty, L. A., & Hodge, J. A. (2015). Mobile application as a prenatal education and engagement tool: A randomized controlled pilot. *Patient Education & Counseling*, *99*(4), 578–582. cin20. https://doi.org/10.1016/j.pec.2015.11.006

Ledford, C. J. W., Womack, J. J., Rider, H. A., Seehusen, A. B., Conner, S. J., Lauters, R. A., & Hodge, J. A. (2017). Unexpected Effects of a System-Distributed Mobile Application in Maternity Care: A Randomized Controlled Trial. *Health Education and Behavior*, *45*(3), 323–330. Scopus. https://doi.org/10.1177/1090198117732110

Lee, T.I., Chiang, Y.H., Guo, J., Chen, M.T., & Chen, Y. (2016). *Dot-it: Managing Nausea and Vomiting for A Peaceful Pregnancy with Personal Pattern Exploration*. In *Proceedings of the 2016 CHI Conference Extended Abstracts on Human Factors in Computing Systems* (pp. 20-25).

Marko, K., Ganju, N., Krapf, J. M., Gaba, N. D., Brown, J. A., Benham, J. J., Oh, J., Richards, L. M., & Meltzer, A. C. (2019). A Mobile Prenatal Care App to Reduce In-Person Visits: Prospective Controlled Trial. *JMIR Mhealth and Uhealth*, *7*(5), e10520. https://doi.org/10.2196/10520

Marko, K. I., Krapf, J. M., Meltzer, A. C., Oh, J., Ganju, N., Martinez, A. G., Sheth, S. G., & Gaba, N. D. (2016). Testing the Feasibility of Remote Patient Monitoring in Prenatal Care Using a Mobile App and Connected Devices: A Prospective Observational Trial. *JMIR Research Protocols*, *5*(4), e200. https://doi.org/10.2196/resprot.6167

Muuraiskangas, S., Mattila, E., Kyttala, P., Koreasalo, M., & Lappalainen, R. (2016). User Experiences of a Mobile Mental Well-Being Intervention Among Pregnant Women. In S. Serino, A. Matic, D. Giakoumis, G. Lopez, & P. Cipresso (Eds.), *Pervasive Computing Paradigms for Mental Health (mindcare 2015)* (Vol. 604, pp. 140–149). https://doi.org/10.1007/978-3-319-32270-4_14

Naughton, F., Cooper, S., Foster, K., Emery, J., Leonardi-Bee, J., Sutton, S., Jones, M., Ussher, M., Whitemore, R., Leighton, M., Montgomery, A., Parrott, S., & Coleman, T. (2017). Large multi-centre pilot randomized controlled trial testing a low-cost, tailored, self-help smoking cessation text message intervention for pregnant smokers (MiQuit): Randomized controlled trial of MiQuit. *Addiction*, *112*(7), 1238–1249. https://doi.org/10.1111/add.13802

Naughton, F., Jamison, J., & Sutton, S. (2013). Attitudes towards SMS text message smoking cessation support: A qualitative study of pregnant smokers. *Health Education Research*, *28*(5), 911–922. https://doi.org/10.1093/her/cyt057

Naughton, F., Prevost, A. T., Gilbert, H., & Sutton, S. (2012). Randomized Controlled Trial Evaluation of a Tailored Leaflet and SMS Text Message Self-help Intervention for Pregnant Smokers (MiQuit). *Nicotine & Tobacco Research*, *14*(5), 569–577. https://doi.org/10.1093/ntr/ntr254

O’Brien, E., Rauf, Z., Alfirevic, Z., & Lavender, T. (2013). Women’s experiences of outpatient induction of labour with remote continuous monitoring. *Midwifery*, *29*(4), 325–331. https://doi.org/10.1016/j.midw.2012.01.014

Payakachat, N., Rhoads, S., McCoy, H., Dajani, N., Eswaran, H., & Lowery, C. (2020). Using mHealth in postpartum women with pre‐eclampsia: Lessons learned from a qualitative study. *International Journal of Gynecology & Obstetrics*, *149*(3), 339–346. https://doi.org/10.1002/ijgo.13134

Platonos, K., Aloysius, A., Banerjee, J., & Deierl, A. (2018). Integrated family delivered care project: Parent education programme. *Journal of Neonatal Nursing*, *24*(1), 29–34. cin20. https://doi.org/10.1016/j.jnn.2017.11.008

Rhoads, S. J., Serrano, C. I., Lynch, C. E., Ounpraseuth, S. T., Heath Gauss, C., Payakachat, N., Lowery, C. L., & Eswaran, H. (2017). Exploring Implementation of m-Health Monitoring in Postpartum Women with Hypertension. *Telemedicine and E-Health*, *23*(10). https://doi.org/10.1089/tmj.2016.0272

Ridgeway, J. L., LeBlanc, A., Branda, M., Harms, R. W., Morris, M. A., Nesbitt, K., Gostout, B. S., Barkey, L. M., Sobolewski, S. M., Brodrick, E., Inselman, J., Baron, A., Sivly, A., Baker, M., Finnie, D., Chaudhry, R., & Famuyide, A. O. (2015). Implementation of a new prenatal care model to reduce office visits and increase connectivity and continuity of care: Protocol for a mixed-methods study. *BMC Pregnancy and Childbirth*, *15*(1), 323. https://doi.org/10.1186/s12884-015-0762-2

Salonen, A. H. (2010). *Effectiveness of an internet-based intervention enhancing Finnish parents’ parenting satisfaction and parenting self-efﬁcacy during the postpartum period*. *Midwifery*, *27*(6), 832-841. https://doi.org/10.1016/j.midw.2010.08.010

Shorey, S., Lau, Ying, Y., Dennis, C.-L., Chan, Y. S., Tam, W. W. S., & Chan, Y. H. (2017). A randomized-controlled trial to examine the effectiveness of the ‘Home-but not Alone’ mobile-health application educational programme on parental outcomes. *Journal of Advanced Nursing*, *73*(9), 2103–2117. https://doi.org/10.1111/jan.13293

Shorey, S., Ng, Y. P. M., Danbjørg, D. B., Dennis, C.-L., & Morelius, E. (2016). Effectiveness of the ‘Home-but not Alone’ mobile health application educational programme on parental outcomes: A randomized controlled trial, study protocol. *Journal of Advanced Nursing*, *73*(1), 253–264. https://doi.org/10.1111/jan.13151

Shorey, S., Yang, Y. Y., & Dennis, C.L. (2018). A Mobile Health App-Based Postnatal Educational Program (Home-but not Alone): Descriptive Qualitative Study. *Journal of Medical Internet Research*, *20*(4), 1–1. cin20. https://doi.org/10.2196/jmir.9188

Soltani, H., Duxbury, A. M. S., Arden, M. A., Dearden, A., Furness, P. J., & Garland, C. (2015). Maternal obesity management using mobile technology: A feasibility study to evaluate a text messaging based complex intervention during pregnancy. *Journal of Obesity*, *2015*. Scopus. https://doi.org/10.1155/2015/814830

Spargo, P., & de Vries, N. K. (2018). ‘Babble’: A smartphone app for parents who have a baby in the neonatal unit: Babble. *Journal of Paediatrics and Child Health*, *54*(2), 121–123. https://doi.org/10.1111/jpc.13817

Strand, A.S., Johnsson B., Hena M., Magnusson, B., & Hallström, I.K. (2021). Developing eHealth in neonatal care to enhance parents’ self-management. *Scandinavian Journal of Caring Sciences*. https://pubmed.ncbi.nlm.nih.gov/33950534/

Tobah, Y. S. B., LeBlanc, A., Branda, M. E., Inselman, J. W., Morris, M. A., Ridgeway, J. L., Finnie, D. M., Theiler, R., Torbenson, V. E., Brodrick, E. M., Meylor de Mooij, M., Gostout, B., & Famuyide, A. (2019). Randomized comparison of a reduced-visit prenatal care model enhanced with remote monitoring. *American Journal of Obstetrics and Gynecology*, *221*(6), 638.e1-638.e8. https://doi.org/10.1016/j.ajog.2019.06.034

Tommasone, G., Bazzani, M., Solinas, V., & Serafini, P. (2016). Midwifery E-Health: From design to validation of “Mammastyle — Gravidanza Fisiologica.” *2016 IEEE 18th International Conference on E-Health Networking, Applications and Services (Healthcom)*, 1–6. https://doi.org/10.1109/HealthCom.2016.7749499

Triebwasser, J. E., Janssen, M. K., Hirshberg, A., & Srinivas, S. K. (2020). Successful implementation of text-based blood pressure monitoring for postpartum hypertension. *Pregnancy Hypertension*, *22*, 156–159. https://doi.org/10.1016/j.preghy.2020.09.001

Valencia, S., Callinan, L., Shic, F., & Smith, M. (2020). Evaluation of the MoMba Live Long Remote Smoking Detection System During and After Pregnancy: Development and Usability Study. *JMIR MHealth and UHealth*, *8*(11), e18809. https://doi.org/10.2196/18809

van der Wulp, N. Y., Hoving, C., Eijmael, K., Candel, M. J., van Dalen, W., & De Vries, H. (2014). Reducing Alcohol Use During Pregnancy Via Health Counseling by Midwives and Internet-Based Computer-Tailored Feedback: A Cluster Randomized Trial. *Journal of Medical Internet Research*, *16*(12), e274. https://doi.org/10.2196/jmir.3493

Whitemore, R., Leonardi-Bee, J., Naughton, F., Sutton, S., Cooper, S., Parrott, S., Hewitt, C., Clark, M., Ussher, M., Jones, M., Torgerson, D., & Coleman, T. (2019). Effectiveness and cost-effectiveness of a tailored text-message programme (MiQuit) for smoking cessation in pregnancy: Study protocol for a randomised controlled trial (RCT) and meta-analysis. *Trials*, *20*(1), 280. https://doi.org/10.1186/s13063-019-3341-4

Wierckx, A., Shahid, S., & Al Mahmud, A. (2014). Babywijzer: An application to support women during their pregnancy. *CHI ’14 Extended Abstracts on Human Factors in Computing Systems*, 1333–1338. https://doi.org/10.1145/2559206.2581179

Willcox, J. C., Campbell, K. J., McCarthy, E. A., Wilkinson, S. A., Lappas, M., Ball, K., Fjeldsoe, B., Griffiths, A., Whittaker, R., Maddison, R., Shub, A., Pidd, D., Fraser, E., Moshonas, N., & Crawford, D. A. (2015). Testing the feasibility of a mobile technology intervention promoting healthy gestational weight gain in pregnant women (txt4two)—Study protocol for a randomised controlled trial. *Trials*, *16*(1). https://doi.org/10.1186/s13063-015-0730-1

Willcox, J. C., Wilkinson, S. A., Lappas, M., Ball, K., Crawford, D., McCarthy, E. A., Fjeldsoe, B., Whittaker, R., Maddison, R., & Campbell, K. J. (2017). A mobile health intervention promoting healthy gestational weight gain for women entering pregnancy at a high body mass index: The txt4two pilot randomised controlled trial. *Bjog-an International Journal of Obstetrics and Gynaecology*, *124*(11), 1718–1728. https://doi.org/10.1111/1471-0528.14552

Yee, L. M., Leziak, K., Jackson, J., Strohbach, A., Saber, R., Niznik, C. M., & Simon, M. A. (2021). Patient and Provider Perspectives on a Novel Mobile Health Intervention for Low-Income Pregnant Women with Gestational or Type 2 Diabetes Mellitus. *Journal of Diabetes Science and Technology*, *15*(5), 1121–1133. https://doi.org/10.1177/1932296820937347

# S3: Behavior Change Theory Use

**Table S2** Behaviour Change Theory Use across Maternity Interventions

|  | Kennelly et al. (2016),(2018) | Krishnamurti et al. (2017) | Himes et al. (2017) | Willcox et al. (2015), (2017) | Soltani et al. (2015)  Hawkins et al. (2019) | Demment et al. (2014) | Moraes et al. (2019) | van der Wulp et al. (2014) | Davis et al. (2018) | Herbec et al. (2014) | Ledford et al. (2015), (2017) | Abroms et al. (2017)  Baruth et al. (2019)  Choi et al. (2015)  Herring et al. (2019)  Naughton et al. (2012),(2013),(2017)  Whitemore et al. (2019) | Harris et al. (2015) |
| --- | --- | --- | --- | --- | --- | --- | --- | --- | --- | --- | --- | --- | --- |
| Behaviour Change Wheel | **X** |  |  |  |  |  |  |  |  |  |  |  |  |
| Behaviour Decision Research Paradigm |  | **X** |  |  |  |  |  |  |  |  |  |  |  |
| Behavioural Economics |  |  | **X** |  |  |  |  |  |  |  |  |  |  |
| CALO-RE Taxonomy of Behaviour Change |  |  |  | **X** |  |  |  |  |  |  |  |  |  |
| Control theory and lifestyle interventions^1^ |  |  |  |  | **X** |  |  |  |  |  |  |  |  |
| Fishbein And Yzer's Integrative Model of Behavior Prediction |  |  |  |  |  | **X** |  |  |  |  |  |  |  |
| Human Centered Design Approach |  |  |  |  |  |  | **X** |  |  |  |  |  |  |
| I-Change Model |  |  |  |  |  |  |  | **X** |  |  |  |  |  |
| Information-Motivation-Behavioural-Skills-Approach |  |  |  |  |  |  |  |  | **X** |  |  |  |  |
| PRIME theory of motivation and addiction |  |  |  |  |  |  |  |  |  | **X** |  |  |  |
| Self-determination Theory |  |  |  |  |  |  |  |  |  |  | **X** |  |  |
| Social Cognitive Theory |  |  |  |  |  |  |  |  |  |  |  | **X** |  |
| Stages of Change Ladder |  |  |  |  |  |  |  |  |  |  |  |  | **X** |

CALO-RE= Coventry, Aberdeen & London – Refined’ taxonomy

^1^Michie, S., Abraham, C., Whittington, C., McAteer, J., & Gupta, S. (2009). Effective techniques in healthy eating and physical activity interventions: a meta-regression. *Health Psychology*, *28*(6), 690.

# S4: Table Showing Missing Commitment and Therapeutic Alliance Attributes

**Table S3**

Studies Missing Commitment and Therapeutic Alliance Attributes

| Commitment | Therapeutic Alliance |
| --- | --- |
| Cotside Laptop System  Bower et al., 2005  Wazz up Mamma  Fontein-Kuipers et al., 2016  Mood Tracking and Alert (MTA) system  Hantsoo et al., 2018 | **Quit4Baby Program**  Abroms et al., 2017  **NICU-2 Home App**  Garfield et al., 2016  **eMoms ROC**  Demment et al., 2014  Graham et al., 2014  **Smart Moms App**  Halili et al., 2018  **Smoking Cessation**  **(SCHB) program**  Harris et al., 2015  **Sleep Self-management Personal health monitor**  Hawkins et al., 2019  **Momba Live Long System Smoking Cessation**  Valencia et al., 2020  **C-Care application**  Xue Chen Ke et al., 2019  Xue Chen Ke et al., 2021 |

Blue Text=Maternity programs; Pink Text= Neonatal programs
